# An efficient multiplier-based FPGA CNN accelerator for Parkinson's disease detection using hand-drawn circle images

**DOI:** 10.3389/frai.2026.1813130

**Published:** 2026-07-17

**Authors:** VedanthSrivatson A., Sivanantham Sathasivam, Prakash Ramachandran

**Affiliations:** School of Electronics Engineering, Vellore Institute of Technology, Vellore, Tamil Nadu, India

**Keywords:** approximate and Karatsuba multipliers, fixed-point quantization (Q4.12), FPGA-based CNN accelerator, hand-drawn circle images, Parkinson's disease detection

## Abstract

**Introduction:**

This study presents a Field-Programmable Gate Array (FPGA)-based convolutional neural network (CNN) accelerator for preliminary Parkinson's disease (PD) handwriting classification using hand-drawn circle images, with emphasis on arithmetic-level optimization through efficient multiplier architectures. Although optimized multipliers have been extensively studied for machine learning acceleration, their application-specific effects on inference consistency and hardware efficiency in healthcare-oriented FPGA implementations remain underexplored.

**Methods:**

To address this issue, a lightweight binary CNN classifier, trained on Google Colab, is deployed on FPGA hardware and evaluated with three multiplier architectures: standard multipliers, approximate logarithmic multipliers, and Karatsuba multipliers. The desktop CNN model was evaluated using both non-augmentation validation and standard augmentation strategies. The primary evaluation methodology used a non-augmentation validation approach, in which augmentation was applied exclusively to the training set, resulting in a software validation accuracy of 92.86%. The standard augmentation strategy achieved a validation accuracy of 97.83% and was used to compare the effects of augmentation before splitting. The trained model was quantized to Q4.12 fixed-point precision and implemented on FPGA hardware, where dense-layer computations were performed using different multiplier architectures. Hardware inference was validated on the NewHandPD hand-drawn circle dataset, and FPGA outputs were compared with software inference results via graphical analysis and Mean Absolute Deviation (MAD) to assess numerical consistency.

**Results:**

Experimental results indicate that the FPGA-based implementation achieved classification behavior closely aligned with software inference while improving hardware efficiency. Under the non-augmentation validation approach, the FPGA implementation achieved 89.73% accuracy compared to 92.86% in software, whereas the standard augmentation strategy achieved 95.40% accuracy compared to 97.83% in software using the Approximate Logarithmic Multiplier.

**Discussion:**

The results indicate the potential feasibility of lightweight CNN deployment with optimized multipliers for resource-efficient edge healthcare applications. However, due to the limited dataset size, the presented findings should be interpreted as a preliminary proof-of-concept study rather than definitive clinical validation.

## 1 Introduction

Field-Programmable Gate Arrays (FPGAs) act as efficient reconfigurable hardware accelerators across applications such as computer vision, deep learning (DL), autonomous systems, Internet of Things (IoT), and biomedical signal processing due to their inherent parallelism, reconfigurability, and low-power operation (Kim and Choi, 2023; Zhou et al., 2024; Yin et al., 2023; Pacini et al., 2023). They support real-time tasks, including image classification, object detection, Unmanned Aerial Vehicle (UAV) navigation, smart surveillance, and classical machine learning (ML)/DL algorithms such as SVM and KNN for embedded and edge applications (Li et al., 2020; Martins et al., 2024; Wang et al., 2022; Abd El-Maksoud et al., 2021). In a convolutional neural network (CNN), recent FPGA architectures also accelerate emerging models like vision transformers and approximate arithmetic units, enabling high-performance and energy-efficient next-generation AI workloads (Garland and Gregg, 2018; Waris et al., 2021; Xia et al., 2021). Despite these extensive advancements, the performance trade-offs between FPGA-based hardware acceleration and CNN implementations have not been studied using an application as a case study. The performance metrics of the FPGA implementation, including area, power, and RTL-level combinational propagation delay, have been reported. Still, the performance of the implemented ML model has not been reported in detail in the existing literature. A thorough comparison of the performance of CNN deployment on an FPGA and on high-performance desktop software is also not available in the literature.

The application of CNNs and other ML models in the biomedical field is vital, and they are widely used to detect and diagnose human diseases from observed clinical and non-clinical information. CNNs are highly useful for classifying Parkinson's disease (PD) from non-clinical observations such as speech, hand-drawn images, and hand tremors. But most of these works demonstrate their results using general-purpose software running on a desktop, and, as mentioned before, hardware-accelerator-based PD detection remains unaddressed, with no existing research demonstrating FPGA-driven implementations for PD classification. In this study, FPGAs are employed as hardware accelerators for ML computation on hand-drawn circle images in PD detection applications owing to their parallel processing capability, hardware resource efficiency, and suitability for energy-efficient edge-oriented inference. The FPGA is interfaced with a data-acquisition module that receives “hand-drawn” circle images from the NewHandPD dataset [47] and accelerates key CNN operations, including convolution, pooling, and dense-layer inference, to enable efficient end-to-end PD detection. To further optimize hardware efficiency in the dense layers, efficient multiplier architectures are incorporated to reduce resource utilization and RTL-level combinational propagation delay while maintaining high classification accuracy. Offloading the CNN detection tasks from the CPU/GPU to optimized FPGA data paths enables real-time, low-power classification suitable for portable and edge-based clinical systems. To the best of the author's knowledge, no prior FPGA accelerator has been designed specifically for this PD detection application, making this study among the first in this domain. In this research, two different multiplier architectures implemented in an FPGA are studied for CNN applications. They are the Approximate Log Multiplier and the Karatsuba Multiplier.

Parkinson's disease is a progressive neurodegenerative disorder marked by tremors, rigidity, and impaired coordination. Early detection remains vital, and ML/DL approaches that analyze “hand-drawn” spirals and circles provide an effective, non-invasive means for accurate PD recognition. Several research studies are carried out to use ML algorithms in detecting PD from hand-drawn datasets (Aldhyani et al., 2024; Alniemi and Mahmood, 2023; Huang et al., 2024; Ianculescu et al., 2024; Pragadeeswaran and Kannimuthu, 2024; Özdemir and Özyurt, 2025; Nithya and Shanmugavadivu, 2025; Farhah, 2024; Shastry, 2025; Al-Jabbar et al., 2025; Hossain and Shorfuzzaman, 2023; Zhao et al., 2023; Mansour, 2024; Agrawal and Sahu, 2024) and more details are given in Section 2.1. As we discussed in this study, an FPGA-based hardware accelerator is implemented for PD detection from hand-drawn circles, and the work is demonstrated using the New HandPD dataset. There are many works that use hardware accelerators (See et al., 2023; Kim et al., 2024; Awais et al., 2023; Aizaz et al., 2023; Foster et al., 2019; Huang et al., 2021; Zaman et al., 2022; Guo et al., 2024; Ullah et al., 2021; Pogue and Nicolici, 2025; Farrukh et al., 2020; Wuraola and Patel, 2022; Derya et al., 2022; Rahman and Bandyopadhyay, 2022; Gupta et al., 2020; Soares et al., 2019; Cao et al., 2015; Chandrasekaran et al., 2025; Elsaid et al., 2024; Vakili et al., 2024; Wu et al., 2021; Yu et al., 2025; Nardes et al., 2026; Salah et al., 2025; Foroumandi et al., 2025; Hu et al., 2025; Gundrapally et al., 2025; Zhao et al., 2026) which are further detailed in Section 2.2. However, there is no literature reporting the use of a hardware accelerator for PD classification using a hand-drawn dataset. One effective method to accelerate ML algorithms is to use optimized multipliers in ML computations. Although several articles discuss the implementation of optimized multipliers for ML hardware acceleration, only a few reports have addressed specific application examples or provided further performance analysis. In this study, a CNN binary classifier is deployed on an FPGA to classify PD from “hand-drawn” circles using various optimized multipliers, and the results are compared with a high-computation desktop approach (Intel i7 processor, 16GB RAM), in which the software model is executed in Google Colab.

To improve data diversity while ensuring reliable model evaluation, two augmentation strategies were considered. The first approach, non-augmentation validation, splits the dataset at the level of the original images and applies augmentation exclusively to the training set, thereby preventing overlap between training and validation samples. This approach is adopted as the primary method for evaluating CNN generalization performance. The second approach, standard augmentation, applies augmentation before dataset splitting, which may lead to optimistic validation accuracy because augmented variants of the same original image appear in both the training and validation sets. However, this method of validation with augmented images helps rigorously evaluate the model's robustness, generalization ability, and performance consistency under real-world variations. Therefore, this method is retained only for comparative methodological analysis. Both approaches were evaluated using the desktop CNN model and corresponding FPGA-based CNN implementation.

In this FPGA-based hardware implementation, three different multipliers are considered: (a) built-in multipliers, (b) approximate logarithmic multipliers, and (c) Karatsuba multipliers. In this process, the CNN model obtained from software training is deployed on an FPGA, and dense-layer computations are performed using various multipliers. The implementation is validated on validation data, and the confidence scores for all validation images are plotted and compared with results from a high-computation desktop approach. The result shows that the CNN implemented on the FPGA performs as well as the CNN executed on the Google Colab platform in the desktop approach. Furthermore, key hardware acceleration metrics, including LUT utilization, FF utilization, DSP utilization, power consumption, power efficiency, and RTL-level combinational propagation delay, are analyzed. The findings reveal that the approximate logarithmic multiplier yields the best optimization across all metrics compared with other multiplier implementations. The findings can be summarized as follows to better appreciate the work.

- The CNN validation accuracy achieved in the high-computation desktop approach (Google Colab) is 92.63% for the non-augmentation validation approach, which serves as the primary evaluation methodology, and 97.83% for the standard augmentation approach retained for comparative analysis of augmentation-before-splitting effects.
- In the FPGA implementation, the best CNN validation accuracy achieved using the approximate logarithmic multiplier is 89.73% under the non-augmentation validation approach and 95.4% under the standard augmentation approach. These results indicate that the FPGA-based CNN performs comparably to the software implementation across both augmentation strategies.
- Significant reductions in LUT utilization (0.43%), FF utilization (0.17%), DSP utilization (0%), and power consumption (3.178 W), together with lower RTL-level combinational propagation delay (151.67 ns), demonstrate the feasibility of a lightweight and resource-efficient FPGA-based CNN implementation for both evaluation methods.

### 1.1 Novel contribution

The methods and experiments that make this study novel and relevant are given below,

1. Implementation of a desktop approach CNN layers for PD classification using hand-drawn circle images and transferring the model to the Xilinx Zynq UltraScale+ ZCU106 FPGA board (xcz7ev-ffvc1156-2-2e) in Vivado Design Suite 2024.1 by saving the Google Colab Q4.12 model file as a (.mem) file and with fixed-point weights and bias quantization.
2. A dual augmentation strategy is introduced to enhance dataset diversity and evaluation reliability. The primary methodology employs a non-augmented validation approach, in which dataset splitting is performed on the original images before augmentation. This ensures a strict separation between training and validation data, preventing overlap between augmented image variants and enabling a more reliable assessment of model generalization for FPGA-based CNN inference. Furthermore, a standard augmentation approach is retained for comparative analysis of augmentation-before-splitting effects.
3. Implementation of a dense layer for Parkinson disease detection using three different multipliers, (1) In-built multiplier, (2) Karatsuba multiplier, and (3) Approximation log multiplier
4. validation of the implemented dense layers using the PD hand-drawn circle validation image dataset by storing the validation image files in the FPGA as (.mem) file and comparing the confidence scores of each image for all multiplier implementations with the confidence score obtained in the desktop approach CNN implementation using graphs and the Mean Absolute Deviation (MAD) method.
5. Hardware acceleration metrics such as LUT utilization, FF utilization, DSP utilization, power consumption, and RTL-level combinational propagation delay were analyzed using the FPGA resource utilization and power analysis reports.
6. The Approximate Logarithmic Multiplier-based dense layer implementation was identified as the most resource-efficient architecture for FPGA acceleration, achieving substantial reductions in LUT utilization, FF utilization, DSP usage, and power consumption while also demonstrating lower RTL-level combinational propagation delay. The FPGA-based CNN implementation achieved a classification accuracy of 89.73% under the non-augmentation validation approach, which serves as the primary evaluation methodology, and 95.40% under the standard augmentation approach for comparative analysis of augmentation-before-splitting effects in PD detection using hand-drawn circle images.

Due to the limited sample size of the NewHandPD dataset (66 original images), the results presented should be interpreted primarily as a proof-of-concept demonstration of the feasibility of CNN-FPGA hardware rather than as definitive clinical validation. Although the proposed system achieved encouraging preliminary classification performance, larger multi-center datasets and broader clinical evaluations are necessary before practical deployment in real-world healthcare environments.

This research is organized so that section 2 reviews related work on PD diagnosis using “hand-drawn” images and FPGA-based multiplier optimization for efficient CNN inference. Section 3 describes the background of the NewHandPD dataset, while Section 4 explains the proposed high-computation-cost desktop CNN architecture for Parkinson's hand-drawing circle detection. Section 5 explores the FPGA hardware implementation of the proposed CNN accelerator, including the conversion flow, the overall accelerator architecture, and the dense-layer design with and without optimized multipliers. Section 6 covers functional verification of the FPGA-based CNN accelerators. Sections 7 and 8 present the results and discussion, and Section 9 concludes the study.

## 2 Related works

Recent research on PD diagnosis and efficient CNN hardware acceleration spans two key directions. The first direction focuses on CNN-based machine learning models that classify motor impairment patterns in handwriting images, such as spirals, waves, and circles. The second category emphasizes reducing LUT, FF, and DSP utilization, RTL-level combinational propagation delay, and power consumption in FPGA-based CNN accelerators through optimized multiplier implementations such as Booth/Karatsuba arithmetic, systolic arrays, and quantization-aware hardware design. Together, these studies highlight parallel advancements in diagnostic accuracy and hardware efficiency, both essential for deployable PD screening systems.

### 2.1 CNN based analysis of “hand-drawn” spirals and circles for PD detection

The following discussion covers the key ideas of the first direction. A layer-by-layer architecture combining DenseNet201 and VGG16 demonstrates strong motor-impairment feature extraction, with DenseNet201 achieving 94% accuracy and 99% ROC, indicating suitability for home-based PD screening (Aldhyani et al., 2024). A CNN binary classifier trained on the balanced NewHandPD and Spiral HandPD datasets achieves 92.33% accuracy and 100% recall, further validating DL's reliability for PD identification (Alniemi and Mahmood, 2023). Transfer learning using VGG16/19, ResNet, and ViT on the NIATS dataset reports up to 96.67% accuracy with VGG19, displaying the effectiveness of pretrained CNNs for early PD diagnosis (Huang et al., 2024). Additional ML solutions, such as frequency-texture analysis of Archimedean spirals, achieve 95.24% accuracy on NeuroPredict AI, presenting a device-free screening alternative (Ianculescu et al., 2024). The PD-specific AI optimizations further boost diagnostic performance. Sine-Cosine Geese Migration Optimization, combined with a Cosine CNN, attains 89.98% accuracy for IoT-based real-time spiral analysis (Pragadeeswaran and Kannimuthu, 2024). ElasticNet-based ViT reaches 99.9% accuracy using optimized feature extraction (Özdemir and Özyurt, 2025). Benchmarking lightweight CNNs identifies GhostNet as the best option for mobile PD detection with 96% accuracy (Nithya and Shanmugavadivu, 2025). Transfer learning using VGG19, InceptionV3, ResNet50v2, and DenseNet169 yields up to 89% accuracy on spiral datasets (Farhah, 2024). A custom CNN-HOG DNN surpasses eleven baselines with strong sensitivity/specificity (Shastry, 2025). A hybrid multi-CNN system combining DenseNet169, MobileNet, VGG16, ACO, and XGBoost achieves 99.3% accuracy and 99.6% sensitivity—one of the highest reported for PD classification (Al-Jabbar et al., 2025). Optimization-driven frameworks dominate PD literature (Hossain and Shorfuzzaman, 2023), including a Spatio-Temporal Siamese Neural Network with memory-augmented Octave-CNN modules for multimodal handwriting analysis (Zhao et al., 2023), and a quantum-optimized QMFOFS-HCNN combining CNN and LSTM to achieve up to 100% accuracy cite mansour2024quantum. A VGG16-BGWO-SVM pipeline achieves 99.8% accuracy on NewHandPD using optimized feature selection (Agrawal and Sahu, 2024). Recently, data-efficient and low-resource learning have emerged as important directions for developing DL models that achieve strong generalization using limited training data. Cao et al. (2025) comprehensively reviewed learning paradigms including few-shot learning, active learning, transfer learning, and geometry-aware optimization for low-resource AI systems, highlighting their importance for resource-constrained applications. Furthermore, Cao et al. (2023) proposed a hyperspherical energy-based active learning framework that improves both representative sample selection and model generalization under limited labeled data. Although these studies focus primarily on improving training efficiency through low-resource and data-efficient learning strategies, the present study complements them by accelerating CNN inference on an FPGA, enabling efficient deployment for practical PD screening in resource-constrained environments.

### 2.2 FPGA-implemented multiplier optimizations for high-performance CNN inference

The key ideas of the second direction are discussed here in the following literature. In parallel with algorithmic progress, the implementation of CNN hardware accelerators has advanced significantly. The following discussion reveals how FPGA implementations accelerate ML computation across different applications. Cryptensor introduces a unified systolic tensor array mapping CNN and NTRU polynomial convolutions onto a single GEMM engine, delivering 22.3% speedup on VGG16 and 95% LUT savings on the XC7Z045 FPGA (See et al., 2023). Iterative logarithmic multipliers further reduce hardware cost and power while preserving ML accuracy, offering a viable arithmetic alternative for edge AI (Kim et al., 2024). FPGA-optimized multiplier designs, such as carry-aware approximate Booth units, achieve 42.9% LUT savings and 53% PDP reduction (Awais et al., 2023), while row-merging approximate multipliers provide 29.26% energy savings with only 0.63% accuracy loss on VGG16. Booth-encoded LUT multipliers (Aizaz et al., 2023) and recursive Karatsuba multipliers (Foster et al., 2019) report up to 70% energy reduction and 43% latency improvement, reinforcing arithmetic-aware CNN optimization. Multi-engine FPGA CNN architectures achieve 98.15% throughput efficiency (1 TOPS) for real-time embedded inference (Huang et al., 2021). MA4C compresses pre-trained CNN weights using MSD encoding, reducing partial sums, and achieving up to 4.2× logic reduction and 1.2× lower multiplier latency for models such as LeNet, MobileNet, and EfficientNet (Zaman et al., 2022). Approximate computing advancements, including LUT-sharing and carry switching multipliers, offer 38.75% power, 17.29% latency, and 28.17% area improvements for error-tolerant imaging tasks (Guo et al., 2024). Lightweight LUT-and-carry chain softcore multipliers outperform DSP-based designs by reducing LUT usage by 53% and delay by 51% (Ullah et al., 2021). Extended Karatsuba matrix multiplication on systolic arrays improves performance per area for DL and homomorphic encryption accelerators (Pogue and Nicolici, 2025). At the system level, TinyYOLO FPGA architectures incorporating Modified Booth Encoding and Wallace trees increase throughput and power efficiency (Farrukh et al., 2020), while square-law activation units for LSTMs eliminate element-wise multipliers without accuracy degradation (Wuraola and Patel, 2022). Multi-scheme post-quantum cryptographic support is enabled through configurable NTT-based polynomial units (Derya et al., 2022). Beyond CMOS, all spin-matrix multipliers provide ultra-low-energy MAC operations for edge AI (Rahman and Bandyopadhyay, 2022). Log_2_-based adaptive quantization replaces multipliers with shift-add operations while preserving accuracy in large DL models (Gupta et al., 2020), and hybrid approximate adders deliver energy savings in image/video pipelines (Soares et al., 2019). Fully homomorphic encryption accelerators using optimized FFT-based and low Hamming weight multipliers achieve 100times speedups for encrypted neural computations (Cao et al., 2015). Hardware-related medical AI also supports this trend. A hybrid ViT-CapsNet FPGA accelerator achieves 99% accuracy with 57% lower processing time for ECG arrhythmia detection (Chandrasekaran et al., 2025). Posit-based multipliers reduce area by 10%, power by 22%, and delay by 43% for ML workloads (Elsaid et al., 2024). DyRecMul dynamically reconfigurable approximate multipliers using CFGLUT5 reduce LUT usage by 64-67% with < 0.3% accuracy loss (Vakili et al., 2024), and an 8-bit LPFP format improves DSP throughput by 1.5× -5× with < 0.5% accuracy degradation for CNN inference (Wu et al., 2021). The FPGA-based neural network accelerators focus on quantization, optimized dataflow, and energy-efficient design. Yu et al. (2025) and Nardes et al. (2026) demonstrate high-throughput and low-latency inference, while Salah et al. (2025) and Hu et al. (2025) use low-precision computation to improve performance, often with higher DSP usage. Additionally, Foroumandi et al. (2025) and Gundrapally et al. (2025) enhance efficiency through architectural and RTL-level optimizations. Beyond Zhao et al. (2026), which explores quantization strategies for lightweight-based models, transformer-based models, and full-chip implementations, demonstrating significant energy reductions.

Together, these studies highlight the growing intersection of non-clinical PD detection with CNN-based approaches, ML, and hardware-efficient DL architectures, underscoring the need for solutions that are both diagnostically accurate and deployable on resource-constrained FPGA platforms. This literature motivates the contributions of the present study.

## 3 Background of dataset

The NewHandPD dataset is a refined, more evenly distributed extension of the original HandPD database developed at Botucatu Medical School, São Paulo State University, Brazil, and curated by researchers at UNESP. It is designed to support PD assessment through handwriting- and drawing-based analysis. The dataset comprises multimodal information, including “hand-drawn” images and dynamic handwriting signals, acquired using a smart digital pen (BiSP). This device records spatial coordinates, pen pressure, and timing information for each writing stroke, enabling detailed characterization of motor impairments associated with PD.

The dataset comprises data from 66 individuals, categorized into two groups:

- Healthy Group: 35 individuals (18 men, 17 women), aged between 14 and 79 years (average 44.05 ± 14.88 years), including 5 left-handed participants.
- Patient Group: 31 individuals (21 men, 10 women), aged between 38 and 78 years (average 57.83 ± 7.85 years), including 2 left-handed participants.

Each participant performed 12 different motor control examinations, such as spiral drawing, meanders, circle drawing, and diadochokinesia(alternating hand movements). Both auditory and visual (image) data were collected from all participants. Consequently, the dataset is well-suited for analyzing motor impairments commonly associated with PD.

**Figure 1:**
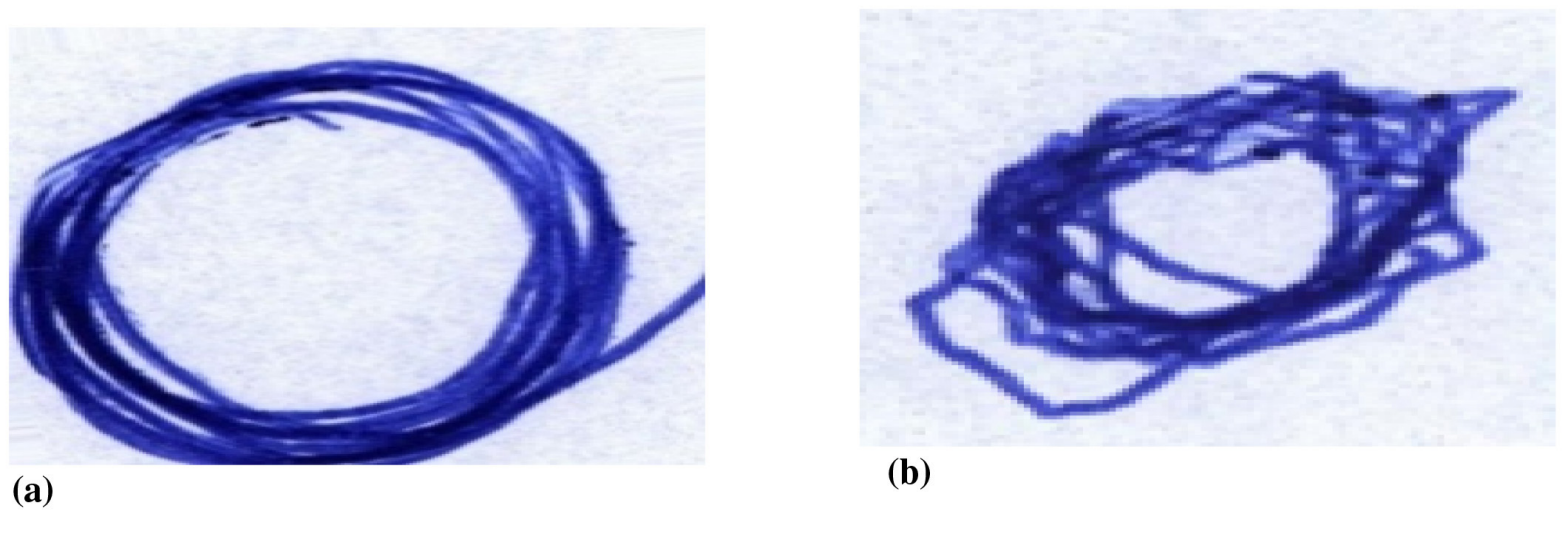
Sample “hand-drawn” circle images used for Parkinson's disease (PD) classification. **(a)** Healthy subject “hand-drawn” circle image. **(b)** Parkinson's disease “hand-drawn” circle image.

In this study, only the circle-drawing task from the NewHandPD dataset was used to train and evaluate the proposed desktop-based CNN model. Representative examples of the circle drawings are shown in Figure 1a for a healthy subject and Figure 1b for a PD patient. These circle images effectively capture key motor characteristics such as drawing accuracy, smoothness, and uniformity, which are critically affected by motor degradation in Parkinson's patients.

The dataset contains one circular image per person, so there are 66 circular images in total, of which 35 are from healthy individuals and 31 are from PD patients. Such a balanced distribution between healthy and patient samples facilitates efficient binary classification and generalization during model training. Therefore, the NewHandPD dataset circle constitutes a lucid, interpretable, and clinically relevant dataset for validating the capacity of DL models to recognize PD from healthy motor patterns using only the “hand-drawn” circle images. Due to its straightforwardness and controlled layout, it is perfect for software-based CNN analysis as well as for hardware-based CNN accelerator implementation on an FPGA platform.

## 4 High-computation desktop approach CNN architecture model for Parkinson's detection

**Figure 2:**
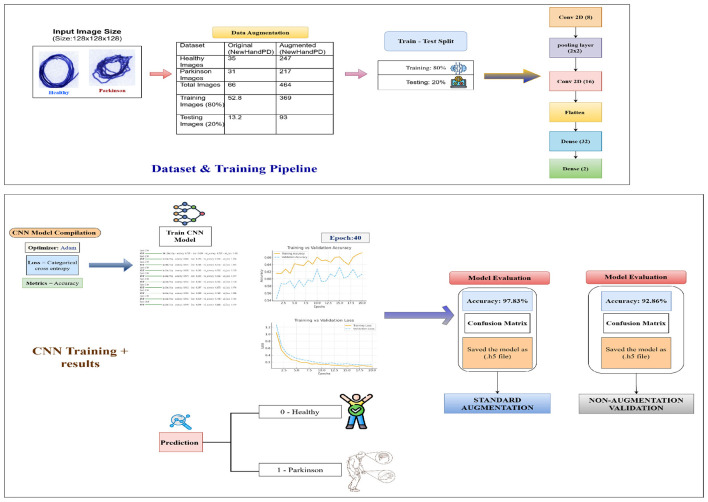
End-to-end desktop approach CNN process flow for Parkinson's disease (PD) detection.

**Figure 3:**
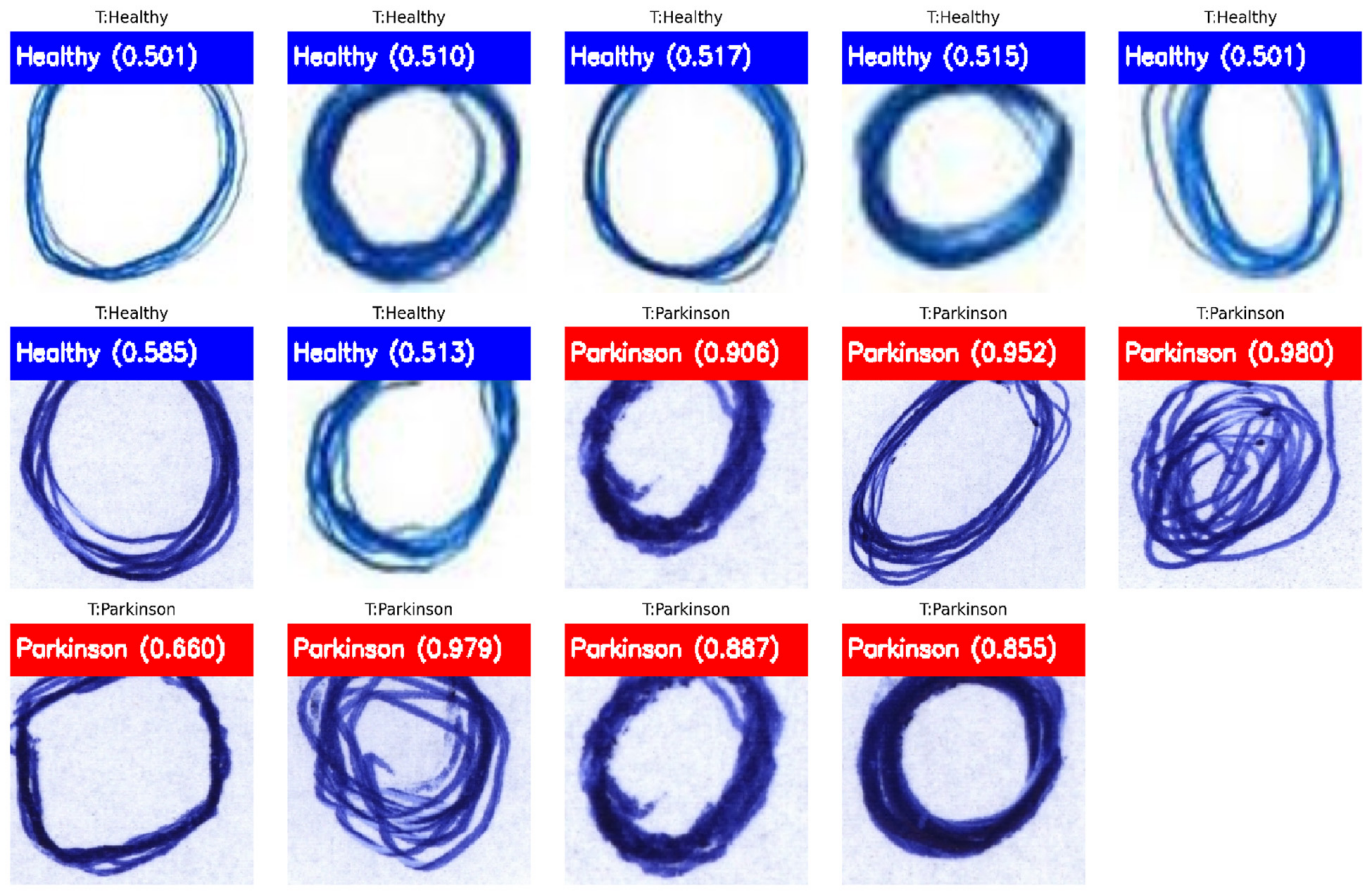
Desktop CNN (Google Colab) predictions with confidence scores for circle images (non-augmentation validation).

**Figure 4:**
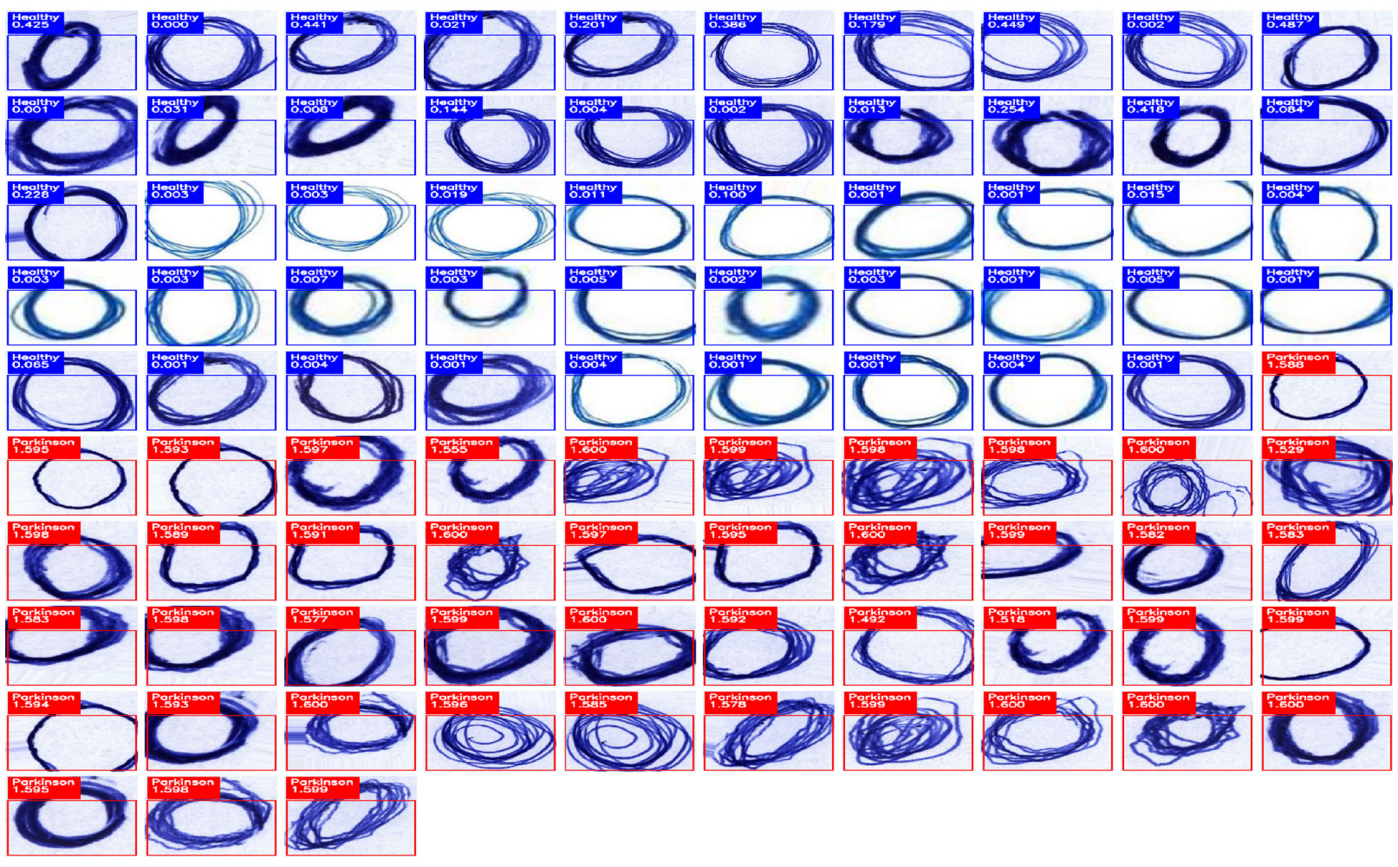
Desktop CNN (Google Colab) predictions with confidence scores for circle images (Standard Augmentation).

The proposed desktop-based CNN was developed and trained using TensorFlow on the Google Colab platform to perform binary classification between Healthy and PD samples derived from the “hand-drawn” circle dataset, Figure 2. The network architecture was intentionally kept compact and hardware-aware to facilitate efficient FPGA deployment without compromising classification performance. The CNN architecture is intentionally designed to be lightweight, with two convolutional layers (8 and 16 filters), to balance classification performance and FPGA resource constraints. To evaluate the impact of data augmentation strategies, two approaches were considered. The primary evaluation methodology employs a non-augmentation validation approach, in which dataset splitting is performed before augmentation to ensure a strict separation between training and validation samples. The PD classification outcomes obtained using the non-augmentation validation approach are illustrated in Figure 3, while those obtained using the standard augmentation approach for comparative analysis of augmentation-before-splitting effects are shown in Figure 4. The detailed network configuration and model hyperparameters are outlined in Tables 1, 2. The architecture includes two convolution-pooling blocks, followed by a flattening stage and two fully connected layers. The first convolutional layer focuses on learning low-level features such as edges and contours, whereas the second layer captures more complex distortions associated with Parkinsonian tremor. The resulting feature vector is then processed by the dense layers to produce the final Healthy vs. PD classification.

**Table 1:** Layer-wise configuration of the desktop CNN model.

Layer	Type	Kernel/neurons	Activation function	Input/output shape
1	Input	28 × 28 × 1	–	28 × 28 × 1
2	Conv2D	3 × 3, 8	ReLU	28 × 28 × 8
3	MaxPool	2 × 2	–	14 × 14 × 8
4	Conv2D	3 × 3, 16	ReLU	14 × 14 × 16
5	MaxPool	2 × 2	–	7 × 7 × 16
6	Flatten	–	–	784
7	Dense	32	ReLU	32
8	Output	2	Softmax	2

**Table 2:** Model hyperparameters.

Parameter	Value
Input image size	128 × 128 × 3
Batch size	8
Learning rate	0.0001
Optimizer	Adam
Epochs	40

Activation Functions:

- ReLU (Rectified Linear Unit):

$$\begin{array}{l} f\left(x\right)=\max\left(0,x\right) \end{array}$$ (1)

Introduces non-linearity as defined in Equation 1 and accelerates training convergence.

- Softmax:

$$\begin{array}{l} f_{i}\left(x\right)=\frac{e^{x_{i}}}{\sum_{j=1}^{n}e^{x_{j}}} \end{array}$$ (2)

The Softmax function converts the final logits into a normalized probability distribution over all classes, where each output represents the likelihood of the input belonging to a particular class, as shown in Equation 2. The final predicted class is obtained using an Argmax operation, which selects the class with the highest probability.

### 4.1 Training results-high-computation desktop approach

The NewHandPD dataset was used to train and evaluate the proposed CNN-based desktop approach. This dataset comprises hand-drawn circle images obtained from both healthy participants and individuals clinically diagnosed with PD. Each drawing captures distinct hand movement patterns that reflect variations in motor coordination, which are commonly affected by PD. The input image size is 128 × 128 was selected based on dataset characteristics to preserve sufficient spatial features while maintaining manageable computational complexity for FPGA implementation. Initially, the dataset contained 66 original images, comprising 35 healthy and 31 Parkinson's samples. A class-balanced validation strategy was adopted by selecting 7 samples from each class (14 in total), rather than a strict 80-20 split, to ensure equal representation of both classes. The remaining 52 images were used for training. Due to the limited size of the NewHandPD dataset, the data was divided into training and validation subsets only, and no separate independent test set was used in this study. Therefore, all reported performance metrics correspond to validation performance. To improve the CNN's generalization capability and reduce overfitting, offline data augmentation techniques were applied. The augmentation operations included rotation, translation (shift), zoom, and horizontal flipping, which simulate realistic variations in drawing orientation, scale, and stroke distribution.

**Figure 5:**
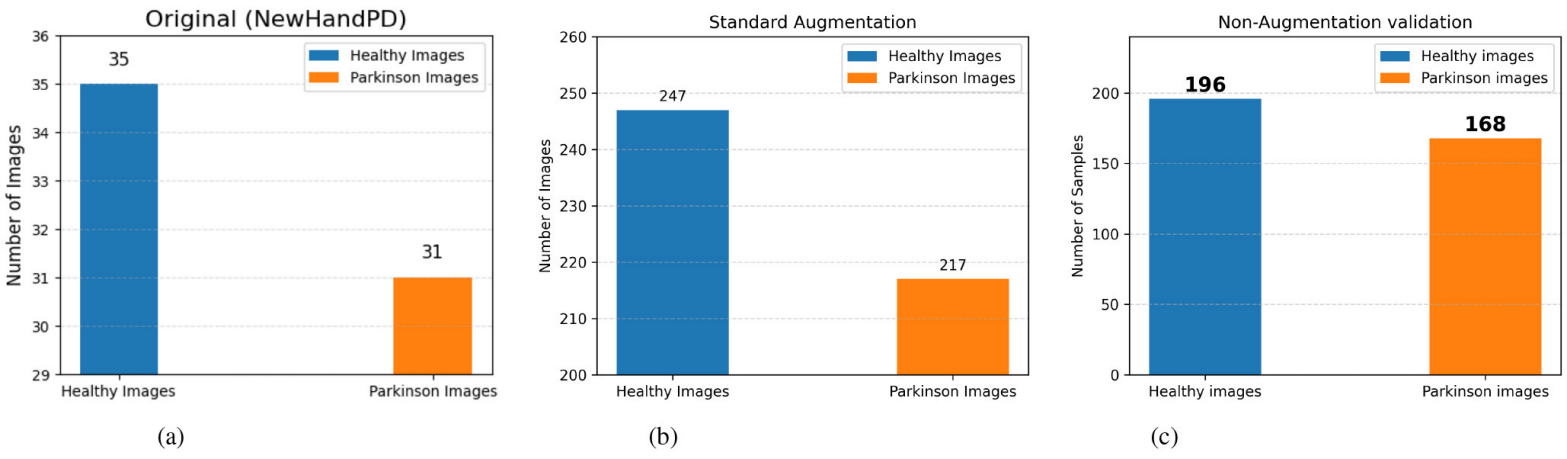
Dataset composition: **(a)** Original dataset, **(b)** standard augmentation, and **(c)** non-augmentation validation.

To investigate the impact of augmentation strategies, two approaches were considered: (1) non-augmentation validation, which serves as the primary evaluation methodology, where the dataset is first split at the original image level and augmentation is applied exclusively to the training set, with no augmentation applied to the validation set, and (2) standard augmentation, where augmentation is applied before dataset splitting in both training and validation. These strategies enable analysis of the effect of data variability on model generalization and provide a basis for evaluating the consistency between software-based and FPGA-based inference. The impact of the original dataset and augmentation strategies is summarized in Table 3. For the non-augmentation validation method, approximately 20% of the isolated images (7 samples per class) are allocated for validation, and the corresponding dataset distribution is presented in Table 4. Representative samples from the dataset are illustrated in Figure 5a.

**Table 3:** Dataset composition under original and standard augmentation.

Dataset	Original (NewHandPD)	standard augmentation (NewHandPD)
Healthy images	35	247
Parkinson's images	31	217
Total images	66	464
Training images	52	369
validation images	14	92

**Table 4:** Dataset distribution for the non-augmentation validation.

Category	Isolated dataset	non-augmentation validation
Healthy images	28 (35–7)	196
Parkinson's images	24 (31–7)	168
Total images	52 (66–14)	378
Training images	52	364
validation images	14	–

#### 4.1.1 Evaluation metrics

To evaluate the classification performance of the proposed CNN model, standard evaluation metrics including Precision, Recall, F1-Score, and Accuracy were employed. These metrics are derived from the confusion matrix, which consists of True Positives (TP), True Negatives (TN), False Positives (FP), and False Negatives (FN). The mathematical expressions used to calculate these evaluation metrics are presented in Equations 3–6.


$$\begin{array}{l} \text{Precision}=\frac{TP}{TP+FP} \end{array}$$ (3)

$$\begin{array}{l} \text{Recall}=\frac{TP}{TP+FN} \end{array}$$ (4)

$$\begin{array}{l} \text{F1-Score}=\frac{2\left(\text{Precision}\times \text{Recall}\right)}{\text{Precision}+\text{Recall}} \end{array}$$ (5)

$$\begin{array}{l} \text{Accuracy}=\frac{TP+TN}{TP+TN+FP+FN} \end{array}$$ (6)

Where *TP*=True Positives (correctly classified samples), *TN*=True Negatives, *FP*=False Positives (incorrectly predicted as positive), and *FN*=False Negatives (missed positive samples).

#### 4.1.2 Non-augmentation validation

To ensure a more reliable evaluation, the primary methodology adopted a non-augmentation validation approach in which the dataset was first split at the original image level into training and validation sets using an 80%–20% ratio. Data augmentation was then applied exclusively to the training set, expanding it to 364 images, while the validation set remained unchanged with 14 original images. The same augmentation techniques—rotation, translation (shift), zoom, and horizontal flipping—were applied, and samples obtained using the non-augmentation validation approach are shown in Figure 5c.

**Figure 6:**
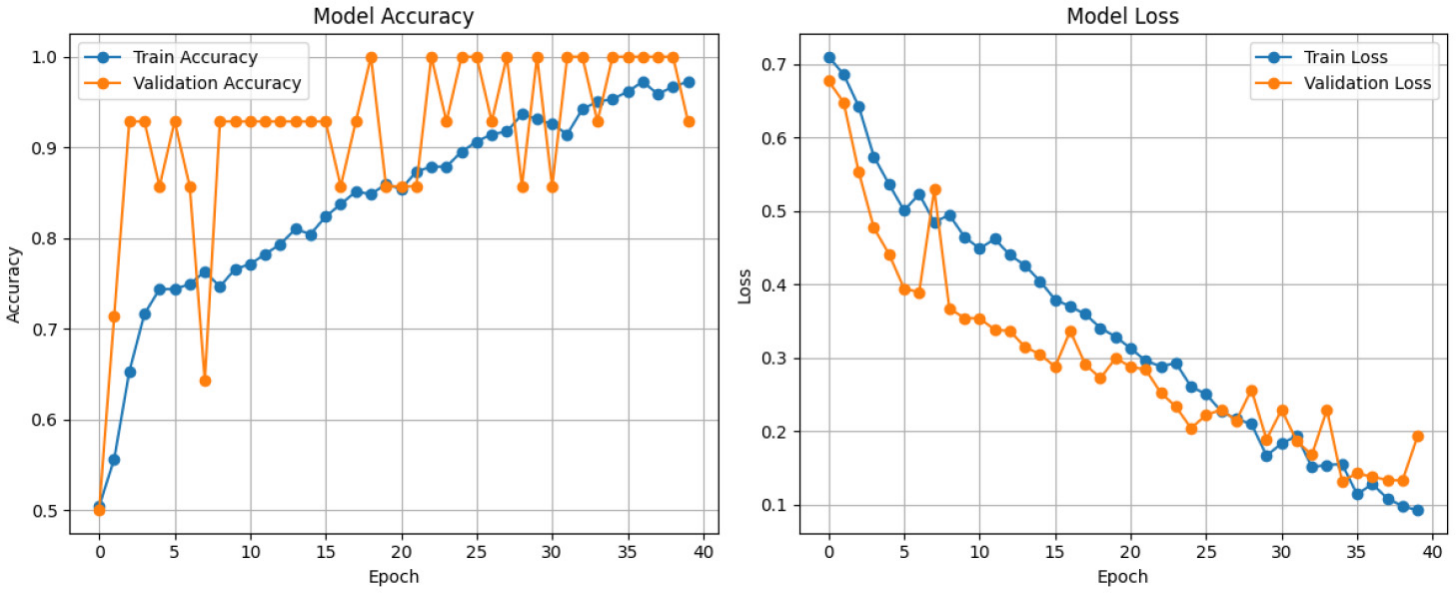
Training and validation accuracy/loss for high-computation desktop approach (non-augmentation validation).

Using this strategy, the CNN achieved a final training accuracy of 97.25% and a validation accuracy of 92.86%, corresponding to 13 correctly classified samples out of 14 validation images. The training process converged within 40 epochs, yielding stable training and validation accuracy and loss curves, as shown in Figure 6. Since augmentation was applied exclusively to the training set after dataset splitting, augmented variants of the same original image were excluded from the validation set, thereby providing a more reliable assessment of model generalization. However, because the validation subset contains only 14 samples, the reported validation accuracy is sensitive to individual misclassifications, with a single prediction error potentially changing it by approximately 7%. Therefore, the reported results should be interpreted with appropriate caution.

#### 4.1.3 Standard augmentation

In the second approach, data augmentation was applied to the dataset before dataset splitting, resulting in a total of 464 images. The augmented dataset was then split 80%-20%, with 369 images allocated to training and 92 to validation. To improve the CNN's generalization capability and mitigate overfitting caused by the limited dataset size, offline augmentation techniques such as rotation, translation (shift), zoom, and horizontal flipping were employed. The samples obtained using the standard augmentation approach are shown in Figure 5b.

**Figure 7:**
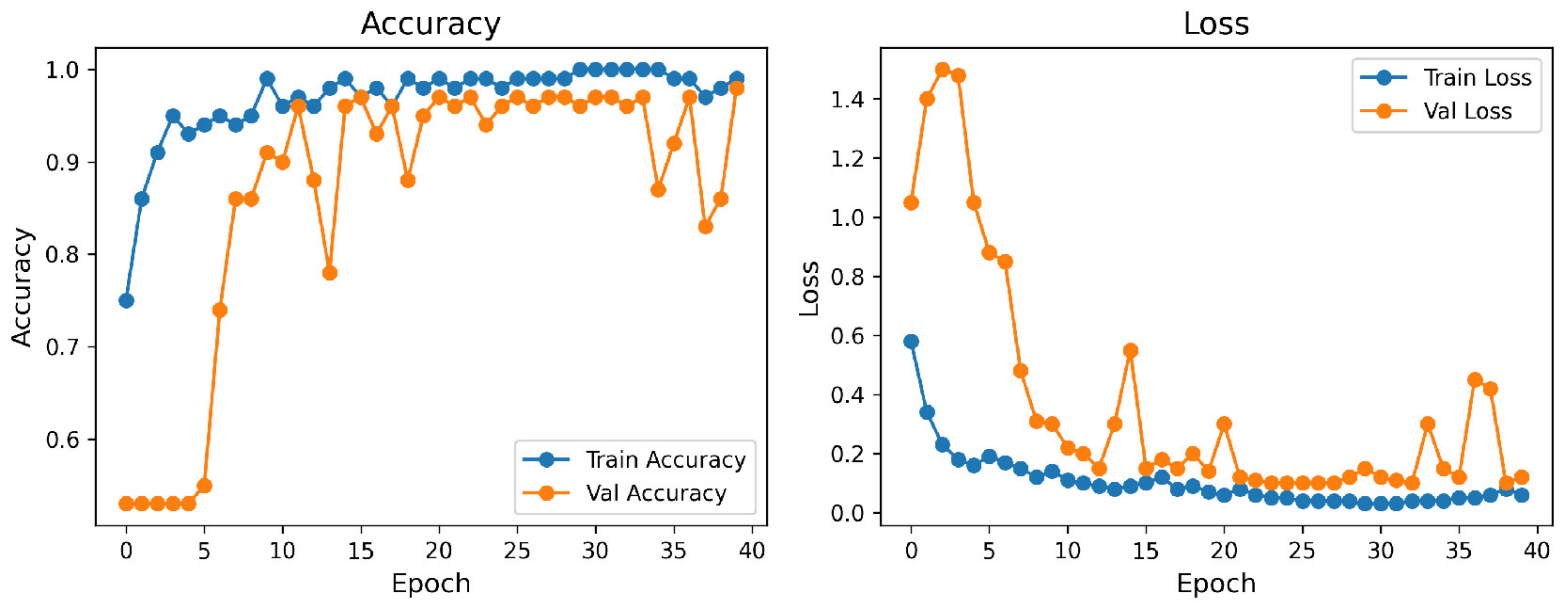
Training and validation accuracy/loss for high-computation desktop approach (standard augmentation).

A 5-fold stratified K-fold cross-validation method was employed to ensure class balance across all folds. In each fold, approximately 80% of the augmented images were used for training and 20% for validation. The CNN model was trained from scratch in each fold, with only pixel normalization applied. The training process converged within 40 epochs, yielding stable training and validation accuracy and loss curves, as shown in Figure 7. The model achieved a training accuracy of 99% and a validation accuracy of 97.83%. However, since augmentation was performed before dataset splitting, augmented variants of the same original image may appear in both the training and validation sets, potentially leading to optimistic estimates of validation accuracy. Therefore, this approach is presented for comparative analysis of augmentation-before-splitting effects rather than as the primary indicator of model generalization performance.

The desktop CNN model achieved a validation accuracy of 92.86% under the non-augmentation validation approach, which serves as the primary evaluation methodology, and 97.83% under the standard augmentation approach used for comparative analysis of augmentation-before-splitting effects.

Using the above equations, the results obtained in Equation 7 are:


$$\begin{array}{l} {\text{Precision}}_{\text{avg}}\approx 0.98,\;{\text{Recall}}_{\text{avg}}\approx 0.98,\;{\text{F1-Score}}_{\text{avg}}\approx 0.98 \end{array}$$ (7)

**Figure 8:**
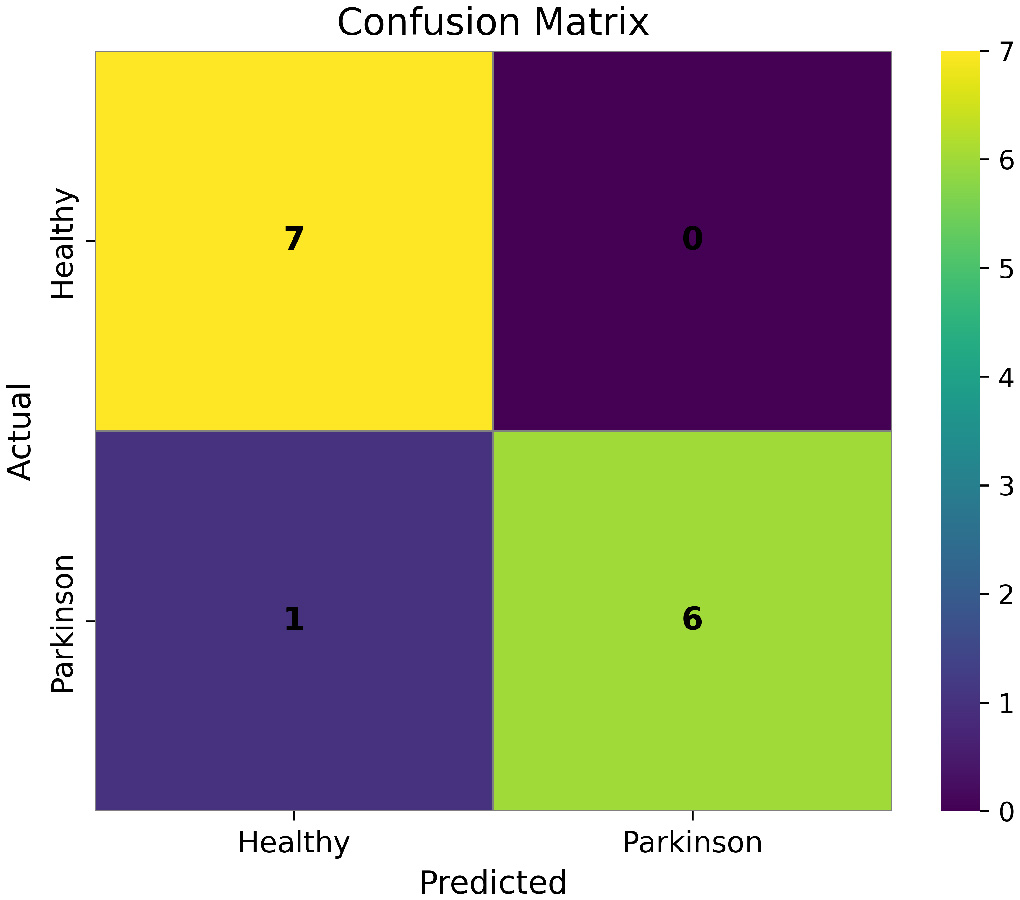
Confusion matrix for high-computation desktop approach CNN model—Parkinson's disease detection (non-augmentation validation).

Under the non-augmentation validation approach, the primary evaluation methodology, the CNN model achieved a validation accuracy of 92.86%, with balanced performance across both classes. The confusion matrix in Figure 8 indicates that seven Healthy and six Parkinson's samples were correctly classified, with only one misclassification. Table 5 corresponds to the precision, recall, and F1-score values, which are approximately 0.94, 0.92, and 0.92, respectively, demonstrating consistent and reliable performance.

**Table 5:** Classification report of high-computation desktop approach for CNN Model (non-augmentation validation).

Predicted class	Precision (%)	Recall (%)	F1-score (%)	Support
0	0.88	1.00	0.92	7
1	1.00	0.86	0.92	7
Accuracy			0.92 (92.86%)	14
Macro Avg	0.94	0.92	0.92	14
Weighted Avg	0.94	0.92	0.92	14

**Figure 9:**
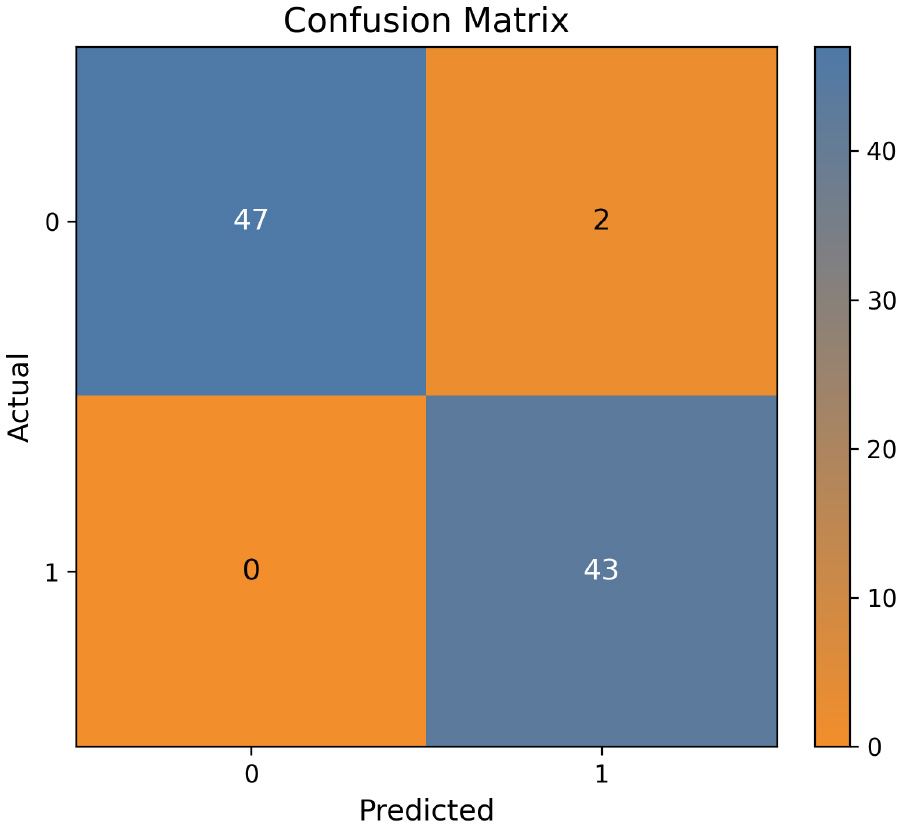
Confusion matrix for high-computation desktop approach CNN model—Parkinson's disease detection (standard augmentation).

Under the standard augmentation approach, both classes exhibit encouraging preliminary classification performance, with precision, recall, and F1-scores all exceeding 0.90, as summarized in Table 6. The confusion matrix in Figure 9 supports these findings: 47 Healthy images and 43 Parkinson's images were correctly classified, while only two samples were misclassified. Although this approach demonstrates high validation accuracy, augmentation performed before dataset splitting may introduce overlap between augmented variants in the training and validation sets, potentially leading to optimistic estimates of validation accuracy.

**Table 6:** Classification report of high-computation desktop approach for CNN model (standard augmentation).

Predicted class	Precision (%)	Recall (%)	F1-score (%)	Support
0	1.00	0.96	0.98	49
1	0.96	1.00	0.98	43
Accuracy			0.98 (97.83)	92
Macro Avg	0.98	0.98	0.98	92
Weighted Avg	0.98	0.98	0.98	92

These results demonstrate that the desktop-approach CNN model can recognize PD with accuracies of 92.86% under the non-augmentation validation approach and 97.83% under the standard augmentation approach, used for comparative analysis of augmentation-before-splitting effects, while maintaining a good balance of precision and recall for both classes under the evaluated experimental conditions. The CNN achieved consistent convergence during both training and validation. Finally, the CNN weights and biases, represented as NumPy arrays, were converted to Q4.12 fixed-point format and stored as (.mem) files for FPGA implementation. The CNN trained on the NewHandPD dataset demonstrates the potential feasibility of lightweight CNN deployment for preliminary Parkinson's handwriting analysis on FPGA platforms. Its compact layer configuration, low parameter count, and fixed-point quantization make it highly suitable for efficient hardware acceleration on FPGA platforms.

## 5 FPGA hardware implementation

The two augmentation methods for a trained desktop CNN model were deployed on an FPGA hardware accelerator to evaluate the performance of various optimized multipliers. The implementation was carried out on the Xilinx Zynq UltraScale+ ZCU106 FPGA board using the Vivado Design Suite 2024.2 (AMD, Santa Clara, CA, USA).

### 5.1 Conversion flow

**Figure 10:**
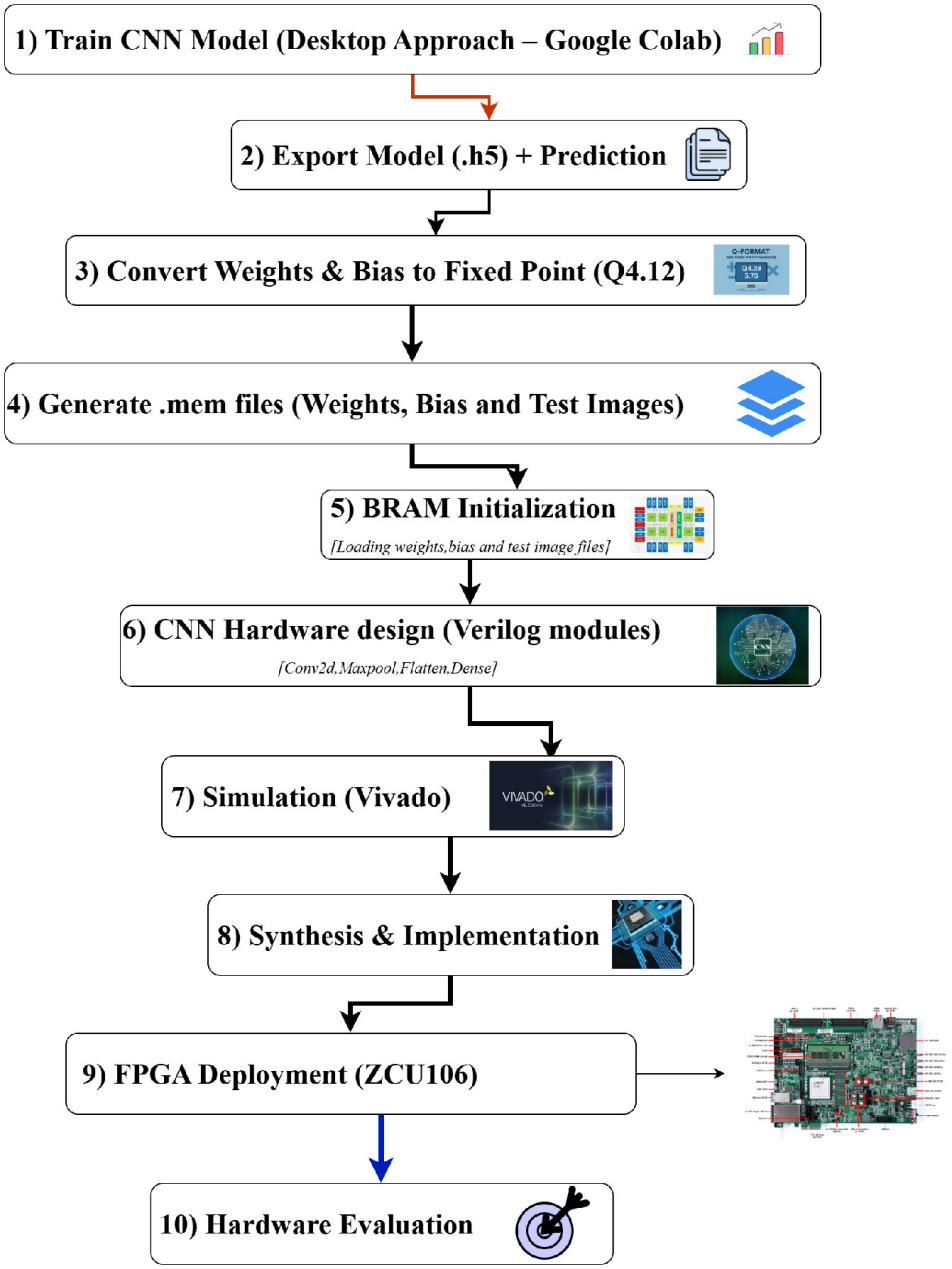
Complete flow process of high-computation desktop approach CNN model to FPGA hardware deployment.

The complete pipeline for deploying CNN software models (.h5) from desktop-based CNN software to FPGA hardware, implemented in Xilinx Vivado Design Suite 2024.2, is shown in Figure 10.

1. Model Training and Export: For the desktop approach, the CNN was trained and validated on Google Colab using two evaluation methods: non-augmentation validation and standard augmentation. The primary methodology employed a non-augmentation validation approach, in which the dataset was first split at the original image level, and augmentation was applied exclusively to the training set. In the standard augmentation approach, data augmentation was applied before dataset splitting, which may lead to optimistic validation accuracy due to overlap between augmented image variants in the training and validation sets. After achieving the targeted accuracy, the model was saved in (.h5) format along with the model summary and prediction results (predicted class and confidence scores).
2. Parameter Extraction: The trained model's weights and biases were extracted layer-wise using NumPy and then converted to a fixed-point representation with Q4.12 quantization to ensure compatibility with FPGA arithmetic.
3. Quantization: To enable efficient FPGA implementation, the model's weights, biases, and activations were quantized from 32-bit floating-point to Q4.12 fixed-point. This was selected to provide a balanced trade-off between numerical precision and hardware resource utilization.
4. Fixed-Point (Q4.12) Representation: The Q4.12 format (Equation 8) consists of 1 sign bit, 3 integer bits, and 12 fractional bits, yielding a numerical range of
$$\begin{array}{l} \left[-2^{3},2^{3}-2^{-12}\right]=\left[-8.0,7.9998\right] \end{array}$$ (8)
  This range was sufficient to represent all the CNN weights, biases, and activation magnitudes observed during training while maintaining minimal quantization error.
$$\begin{array}{l} x_{\text{fixed}}=\mathrm{round}\left(x_{\text{float}}\times 2^{12}\right) \end{array}$$ (9)
  Each floating-point value was converted to its fixed-point form using Equation 9. Reverse scaling during inference was performed implicitly through arithmetic normalization when reconstructing the confidence outputs.
  To justify the selection of the Q4.12 format, a comparison in Table 7 was made with Q2.14 and Q8.8 representations, which differ in dynamic range and precision.
  The Q2.14 offers higher precision but a limited dynamic range, which can lead to overflow during accumulation. Q8.8 provides a larger range but reduced precision, increasing quantization error. Q4.12 achieves a balanced trade-off, ensuring sufficient range and accuracy, as evidenced by the low MAD values observed between software and hardware outputs. Therefore, Q4.12 was selected as the most suitable representation for the proposed FPGA-based CNN accelerator.
5. Quantization-Aware Validation: Before deployment, the quantized models for both approaches were revalidated on a desktop CNN (Google Colab) to ensure numerical stability under fixed-point arithmetic. The Q4.12 representation preserved the original model accuracy with minimal degradation, confirming suitability for FPGA implementation. After validation, the quantized weights, biases, and validation images were finalized for hardware use.
6. Memory File Generation: Following validation, the finalized quantized parameters were formatted into (.mem) files required for BRAM initialization in the FPGA design. The weights and biases of each CNN layer, along with the validation images—14 for the non-augmentation validation approach (Primary method) and 92 for the standard augmentation approach—were converted into address-mapped Q4.12 (.mem) files, structured according to Vivado and Verilog module specifications.
7. Verilog Integration and Simulation: The quantized memory (.mem) files were integrated into corresponding Verilog modules for each CNN layer (Conv2D, MaxPool, Flatten, Dense1, Dense2). A top-level controller was developed using Finite State Machine (FSM) control to manage layer execution and BRAM data flow.
8. Synthesis and Implementation: Using Xilinx Vivado, the design was synthesized and implemented to obtain resource utilization and power reports.
9. Bitstream Generation and Hardware validation: The synthesized design was exported as a (.bit) file and programmed into the ZCU106 board. FPGA-based inference was performed on all validation images (14 for the non-augmentation validation approaches and 92 for the standard augmentation), producing class predictions and confidence scores that were compared with those from the desktop CNN approach.

**Table 7:** Comparison of fixed-point formats.

Format	Integer bits	Fractional bits	Key trade-off
Q2.14	2	14	High precision, limited range (overflow risk)
Q4.12	4	12	Balanced range and precision
Q8.8	8	8	Large range, lower precision

### 5.2 Top-level CNN accelerator design

**Figure 11:**
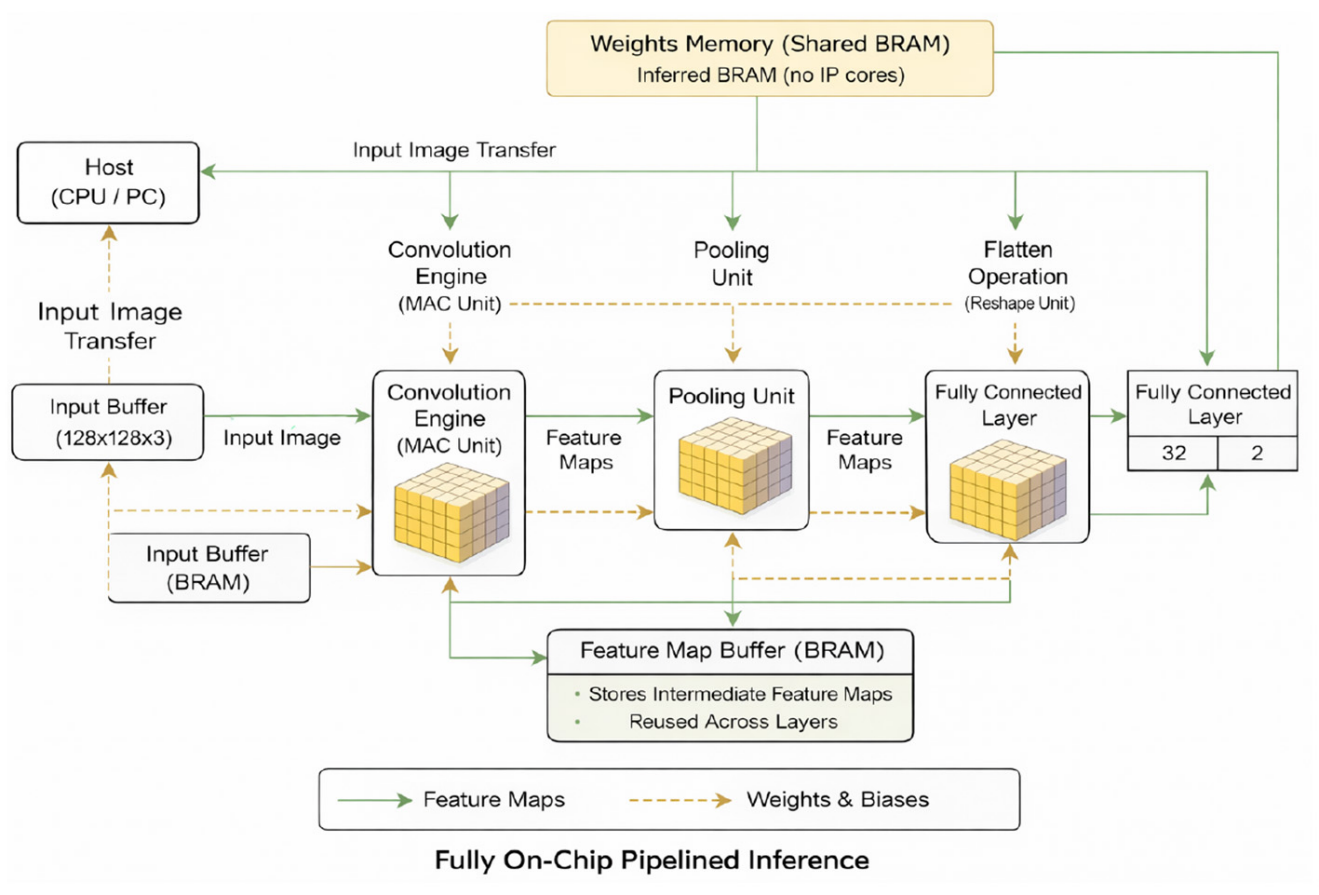
Overall dataflow and interconnection between processing units and on-chip memory.

The top-level architecture of the FPGA CNN accelerator consists of Verilog HDL blocks representing each CNN layer operation, as shown in Table 8. The overall dataflow and interconnection between processing units and on-chip memory are illustrated in Figure 11. These modules are coordinated by a Finite State Machine (FSM) controller, which manages layer timing, synchronization, and BRAM I/O control. Each layer is implemented as an independent Verilog module, allowing section-wise validation, synthesis, and hardware reuse. Data between layers is transferred via dual-port BRAMs, ensuring continuous pipelined processing without external memory access. Among these modules, the Dense Layers (dense1 and dense2) are the most computationally intensive, as they contain the majority of the multiply-accumulate (MAC) operations. Therefore, they are further optimized using built-in, approximate logarithmic, and Karatsuba multipliers, which are discussed in detail in Section 5.3.

**Table 8:** Top-level architecture of the FPGA CNN accelerator-Verilog layer blocks representing each CNN operation.

S.No	Module name	Verilog layer description
1	conv2d_layer.v	Performs convolution operations using fixed-point multiplications to extract spatial features.
2	maxpool_layer.v	Extracts dominant features using a 2 × 2 max-pooling operation.
3	flatten_layer.v	Converts multi-dimensional convolutional feature maps into a one-dimensional vector.
4	dense1 (784 × 32).v	Implements the first fully connected layer with selectable multiplier architectures.
5	dense2 (32 × 2).v	Implements the final fully connected output layer for binary classification.
6	argmax.v	Computes the final classification by identifying the neuron with the highest confidence score.
7	top_cnn_accel.v	The FSM controller manages BRAM read/write operations, layer-enable signals, and overall data flow.
8	weightloader.v	Loads pre-trained CNN weights and biases into on-chip BRAMs during system initialization.
9	imageloader.v	Loads input images into BRAM and converts pixel values into fixed-point format for processing.

### 5.3 Dense layer architecture

The dense (fully connected) layers represent the most computationally intensive stage of the CNN accelerator, performing the majority of multiply-accumulate (MAC) operations. To enhance performance and energy efficiency, three different multiplier architectures—In-built, Approximate Logarithmic, and Karatsuba were developed and integrated into the FPGA-based CNN accelerator.

Two versions of the dense-layer architecture multipliers were implemented:

1. Dense Layer without optimized multipliers - using the In-built multiplier as the baseline LUT + adder design that performs multiplication using shift-add logic without DSP resources.
2. Dense Layer with optimized multipliers - implemented using an integrated Hybrid Multiplier module that contains both the Approximate Logarithmic Multiplier (ALM) and the Karatsuba multiplier.

The top-level module selects which multiplier to activate based on the mode signal from the testbench. Both designs operate on fixed-point (Q4.12) activations and weights stored in BRAM.

#### 5.3.1 Dense layer without optimized multipliers (in-built multipliers)

**Figure 12:**
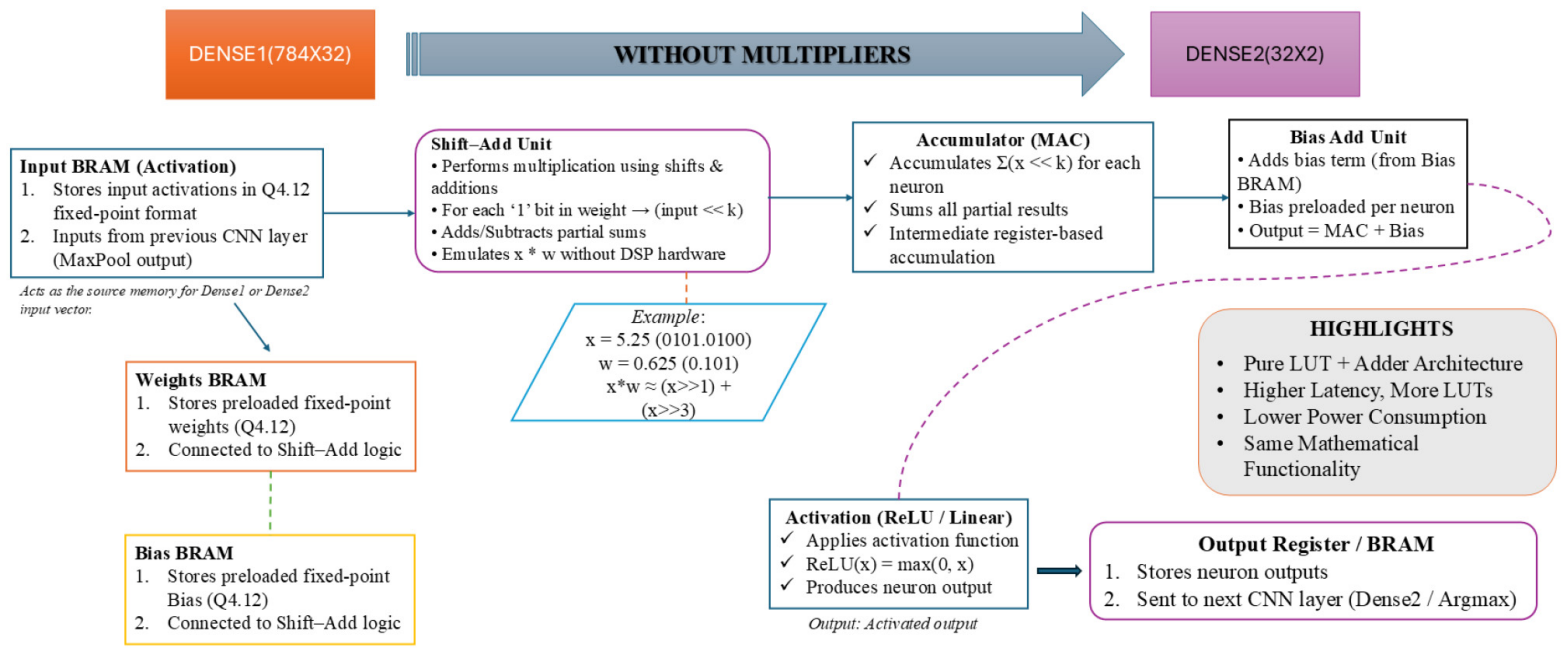
Baseline dense layer architecture (in-built multipliers).

The baseline dense layer employs the built-in Verilog (*) operator to perform exact fixed-point multiplication using DSP48 blocks, and the design ensures bit-accurate computation equivalent to the desktop approach CNN model and serves as the reference architecture in Figure 12, for all hardware comparisons.

Each dense layer reads activations and weights from BRAM, performs MAC operations, adds bias, applies the ReLU activation function, and writes results back to BRAM for the next stage. This LUT-adder architecture eliminates the need for DSP blocks while reducing power consumption, at the cost of a slightly higher RTL-level combinational propagation delay. The arithmetic unit uses DSP48-based exact arithmetic, providing the highest precision but consuming more DSPs, LUTs, and power. The key features of this approach include exact arithmetic computation using DSP48 blocks and a straightforward fixed-point Verilog implementation, making it easier to integrate into existing CNN architectures. However, the system design also poses a few problems, leading to high DSP and LUT utilization, which limits the scalability of deploying deeper or more complex CNN models. Power consumption increases due to continuous DSP switching activity. Furthermore, the design is not suitable for FPGA accelerators operating under strict resource and power constraints. In the Dense (784 × 32) and Dense (32 × 2) layers, instantiate an In-built Multiplier module separately, with the multiplier configuration synthesized and evaluated independently to enable real-time hardware evaluation and a fair comparison across different multiplier configurations.

#### 5.3.2 Dense layer architecture with optimized multipliers

**Figure 13:**
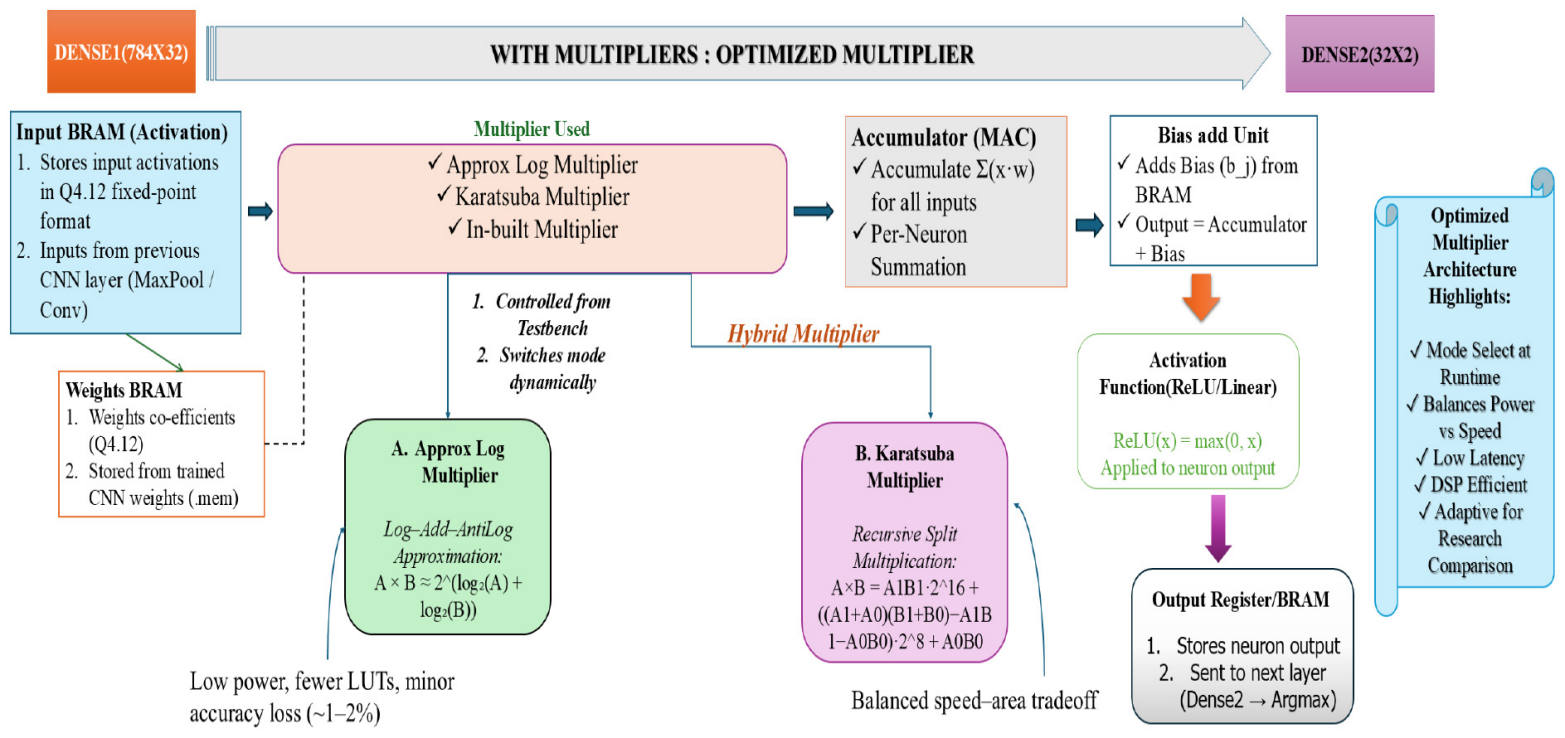
Optimized dense layer architecture (multipliers integration).

The optimized multipliers are incorporated into the dense layers to improve hardware efficiency and computational performance. Specifically, two optimized multiplier architectures—(1) the Approximate Logarithmic Multiplier (ALM) and (2) the Karatsuba Multiplier—are integrated into a Hybrid Multiplier Unit. The selected multiplier architectures enable evaluation of the trade-off between computational accuracy and hardware resource utilization, particularly in the context of approximate computing. The testbench dynamically selects the active multiplier via FSM-based mode-control signals, and the complete operational flow is illustrated in Figure 13. Each dense layer operates on fixed-point Q4.12 data, receiving inputs from the preceding CNN layer, performing multiply-accumulate (MAC) operations, applying bias and activation functions, and storing the computed outputs in BRAM for subsequent processing. The Dense (784 × 32) and Dense (32 × 2) layers use a hybrid multiplier architecture based on Approximate Logarithmic Multiplier (ALM) and Karatsuba multiplier modules to improve performance. The selection of the multiplier is controlled by the testbench, enabling real-time hardware evaluation of the CNN accelerator under different multiplier configurations.

**Figure 14:**
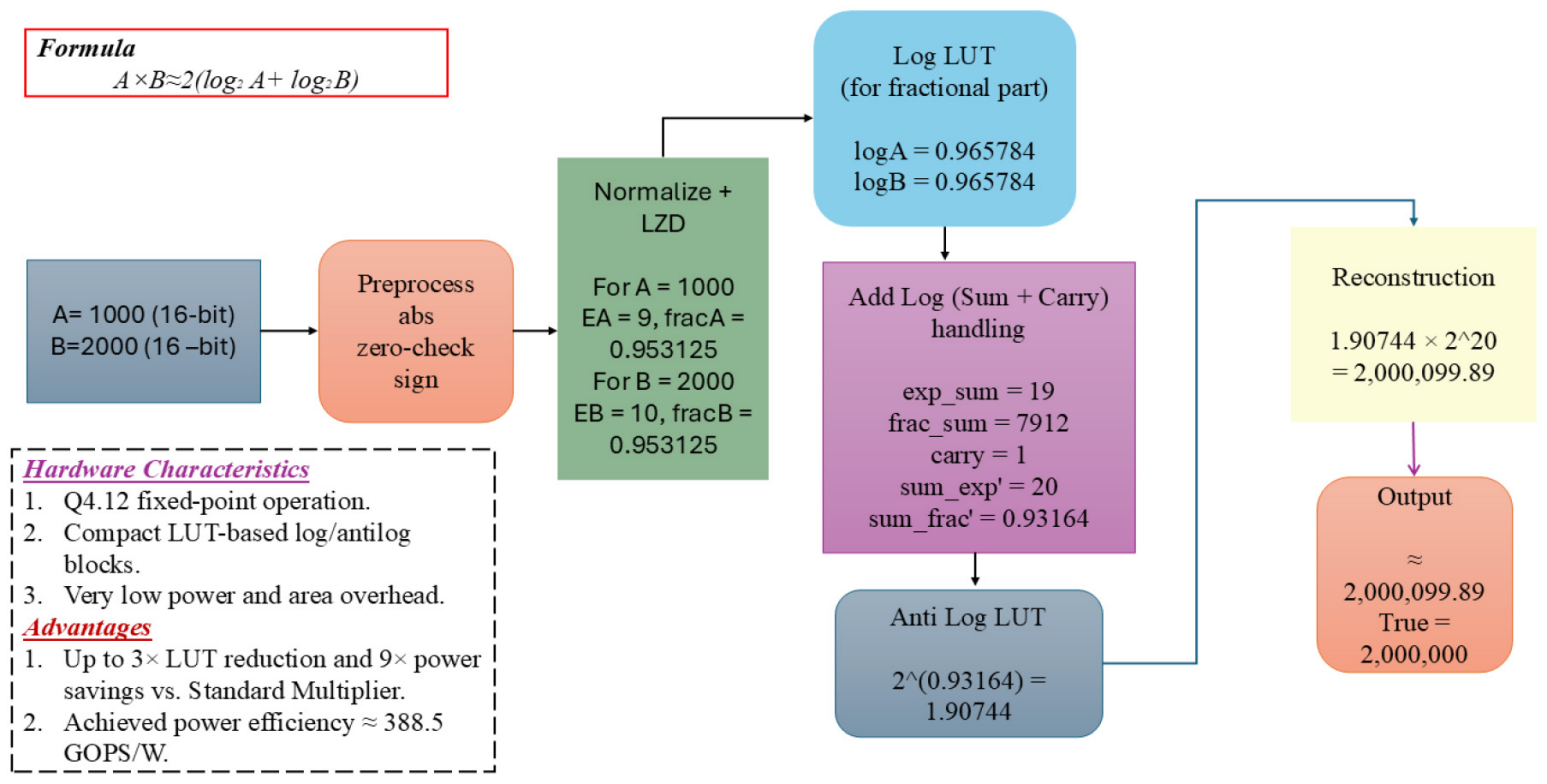
Operational flow of the approximate logarithmic multiplier (16-bit).

**Figure 15:**
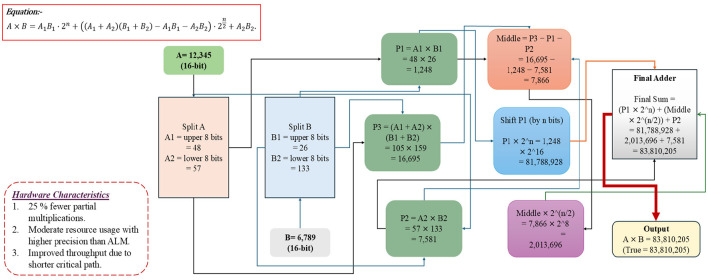
Operational flow of Karatsuba multiplier (16-bit).

1. Approximate Logarithmic Multiplier
  The Approximate Logarithmic Multiplier (ALM) replaces conventional multiplication with a low-cost logarithmic addition + antilog reconstruction flow. The proposed 16-bit ALM utilizes LUT-based log/antilog computation and reconstructs data using only shift and LUT-based adder operations. As illustrated in Figure 14, the formula-based, real-time approach eliminates the need for hardware multipliers, thereby avoiding the use of DSP48 and significantly reducing overall DSP resource utilization.
2. Karatsuba Multiplier
  The Karatsuba Multiplier uses a recursive divide-and-conquer technique to minimize the number of partial products. For two n-bit inputs:
  In Figure 15, the 16-bit Karatsuba multiplier implementation is shown; the operands are divided into two 8-bit halves, and three partial multiplications are recursively combined to obtain the result. The selected multiplier architectures enable evaluation of the trade-off between computational accuracy and hardware resource utilization, particularly in the context of approximate computing.

## 6 FPGA hardware functional verification

The functional verification of the FPGA-based CNN accelerator was achieved using a dedicated Verilog testbench. The purpose of this phase was to verify the implementation of each CNN layer, confirm the FSM-based control flow, and verify agreement between the predicted outputs and those of the reference desktop-based CNN model. All 14 validation images (non-augmented) and 92 validation images (Standard Augmentation approach), pre-processed and quantized to Q4.12 fixed-point format, were loaded one by one into on-chip Block RAM (BRAM) and passed through the accelerator pipeline. The testbench is responsible for producing all the necessary control signals, clock, reset, start, and load weights, while also monitoring the done flags, intermediate outputs, and final classification results from the top CNN accelerator module. The CNN hardware model replicated the same network structure used in the desktop approach CNN (Google Colab) implementation, comprising on Conv2D (28 × 28 × 1 → 28 × 28 × 8), MaxPool (28 × 28 × 8 → 14 × 14 × 8), Conv2D (14 × 14 × 8 → 14 × 14 × 16), MaxPool (14 × 14 × 16 → 7 × 7 × 16), Flatten (7 × 7 × 16 → 784), Dense1 (784 × 32) and Dense2 (32 × 2). Each layer module was tested to confirm accurate data propagation and correct loading of weights and biases from the quantized .mem files. This verification covers timing consistency, ensuring that the finite-state machine (FSM) handles synchronization without glitches or stalls. The testbench verification confirms that all read and write sequences are executed in the intended order. Layer enable signals toggled precisely on schedule, and data handoff between layers ran seamlessly. For every input image, the FPGA hardware accelerator produced two key outputs:

1. Predicted Class: 0 = Healthy,1 = Parkinson
2. Confidence Score:Argmax probability corresponding to the predicted class.

The hardware-generated predictions were compared with those obtained from the desktop-based CNN model (Google Colab) to confirm functional equivalence and the correctness of the computation after quantization.

### 6.1 Mean Absolute Deviation (MAD) analysis

The numerical deviation between software and hardware confidence scores was quantified using the Mean Absolute Deviation (MAD), which provides a direct measure of the average absolute difference between corresponding inference outputs and is expressed in Equation 10:


$$\begin{array}{l} \text{MAD}=\frac{1}{N}\overset{N}{\underset{i=1}{\sum }}|C_{\text{HW},i}-C_{\text{SW},i}| \end{array}$$ (10)

where *C*_*HW,i*_ and *C*_*SW,i*_ denote the confidence scores of the *i*-th validation image obtained from the FPGA hardware implementation (using the Approximate Logarithmic or Karatsuba multiplier) and the desktop CNN implementation (Google Colab), respectively. Here, *N* = 14 for the non-augmentation validation approach, which serves as the primary evaluation methodology, and *N* = 92 for the Standard Augmentation approach used for comparative analysis of augmentation-before-splitting effects.

It is important to clarify that two different augmentation strategies were evaluated in this study, leading to different MAD values: (i) non-augmentation validation, where augmentation was applied exclusively to the training set (*N* = 14), and (ii) Standard Augmentation, where augmentation was applied before dataset splitting (*N* = 92).

The previously reported MAD value (≈0.2595) corresponds to the non-augmentation validation approach, where the smaller validation dataset size and differences in the distribution between training and validation samples result in a relatively higher numerical deviation.

Conversely, the MAD values reported in Table 10 (0.114038 and 0.115260) correspond to the Standard Augmentation approach, where augmentation was applied before dataset splitting, resulting in closer numerical agreement between software and hardware outputs. Therefore, the observed variation in MAD values arises from differences in augmentation strategy and dataset size across the two experimental settings.

#### 6.1.1 Non-augmentation validation

**Figure 16:**
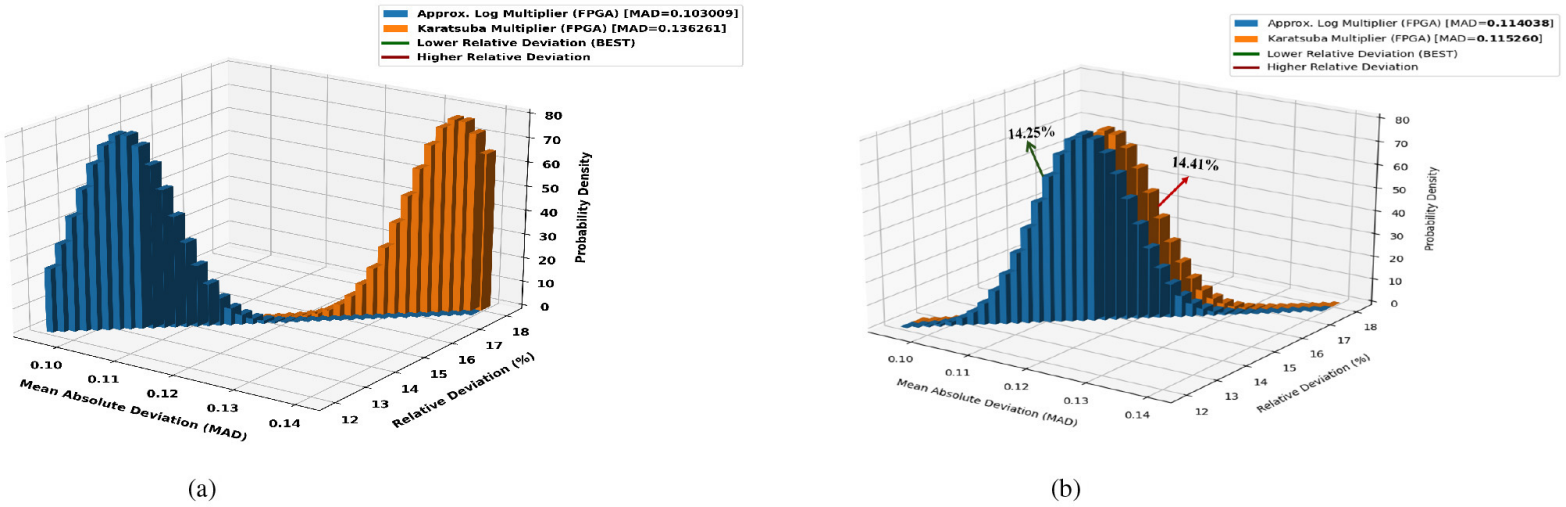
Mean Absolute Deviation (MAD) and relative deviation distribution between the desktop CNN (Google Colab) and FPGA-based CNN implementations under **(a)** non-augmentation validation [Primary method] **(b)** Standard Augmentation.

For the non-augmentation validation approach (*N* = 14), which serves as the primary evaluation methodology, the results shown in Table 9 and Figure 16a reveal a similar trend, with the FPGA-based CNN maintaining close agreement with the desktop model. The Approximate Logarithmic Multiplier achieves a lower MAD (0.103009) and relative deviation (10.30%) compared to the Karatsuba multiplier (MAD = 0.136269, relative deviation = 13.63%). The deviation distributions further indicate a clearer separation between the two multipliers, with the approximate multiplier providing more stable and consistent confidence outputs.

**Table 9:** Mean Absolute Deviation (MAD) between desktop CNN (Google Colab) and FPGA-based CNN predictions (Non-augmentation validation).

Software vs. hardware modes	MAD	Rel. dev. (%)
Desktop CNN vs. Approx. log multiplier (FPGA)	0.103009	10.30
Desktop CNN vs. Karatsuba multiplier (FPGA)	0.136269	13.63

Overall, across both approaches, the Approximate Logarithmic Multiplier consistently achieves better numerical alignment with the desktop CNN outputs than the Karatsuba multiplier. The observed accuracy degradation (2.4%) is primarily due to fixed-point quantization. At the same time, the contribution of approximate multiplication to the overall error remains limited, as evidenced by the low MAD values and the small difference between multiplier implementations. These results confirm that the proposed FPGA-based CNN accelerator achieves inference performance that closely matches the software baseline, with minimal deviation, while benefiting from reduced hardware complexity, power consumption, and an efficient fixed-point implementation.

Therefore, MAD is used as a quantitative metric to assess the numerical consistency between software- and FPGA-based inference outputs. Simulation waveforms verified the expected timing relationships between control and data signals, demonstrating that the FSM controller effectively orchestrated layer execution without data hazards or dead cycles.

This testbench-based verification established the correctness of the complete CNN accelerator before FPGA synthesis. It ensured that subsequent hardware results could be directly correlated with software outputs during experimental evaluation. The hardware-generated predictions were evaluated for both augmentation strategies to ensure consistent functional behavior across different data preparation approaches.

#### 6.1.2 Standard augmentation

For the Standard Augmentation approach (*N* = 92), the results presented in Table 10 and illustrated in Figure 16b demonstrate that both FPGA hardware implementations closely follow the prediction confidence trends of the desktop CNN. The MAD values of the two hardware designs differ by only about 0.0012, indicating a strong correlation between software and hardware inference outputs. The Approximate Logarithmic Multiplier achieves a slightly lower MAD (0.114038) and relative deviation (14.25%) compared to the Karatsuba multiplier (MAD = 0.115260, relative deviation = 14.41%). This indicates better numerical consistency of the approximate multiplier with the software CNN.

**Table 10:** Mean Absolute Deviation (MAD) between desktop CNN (Google Colab) and FPGA-based CNN predictions (standard augmentation).

Software vs. hardware modes	MAD	Rel. dev. (%)
Desktop CNN vs. Approx. log multiplier (FPGA)	0.114038	14.25
Desktop CNN vs. Karatsuba multiplier (FPGA)	0.115260	14.41

## 7 Results

### Hardware and software validation for PD detection using hand-drawn circles

The hardware-software correlation of the FPGA-based CNN accelerator was evaluated using 14 (non-augmentation validation) and 92 (Standard Augmentation) and quantized validation images under two optimized multiplier configurations.

**Figure 17:**
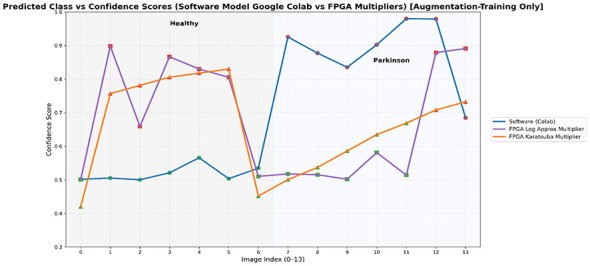
Comparison of predicted class confidence scores between the desktop approach CNN (Google Colab) and FPGA Hardware implementations using Approximate Logarithmic and Karatsuba multipliers (non-augmentation validation).

**Figure 18:**
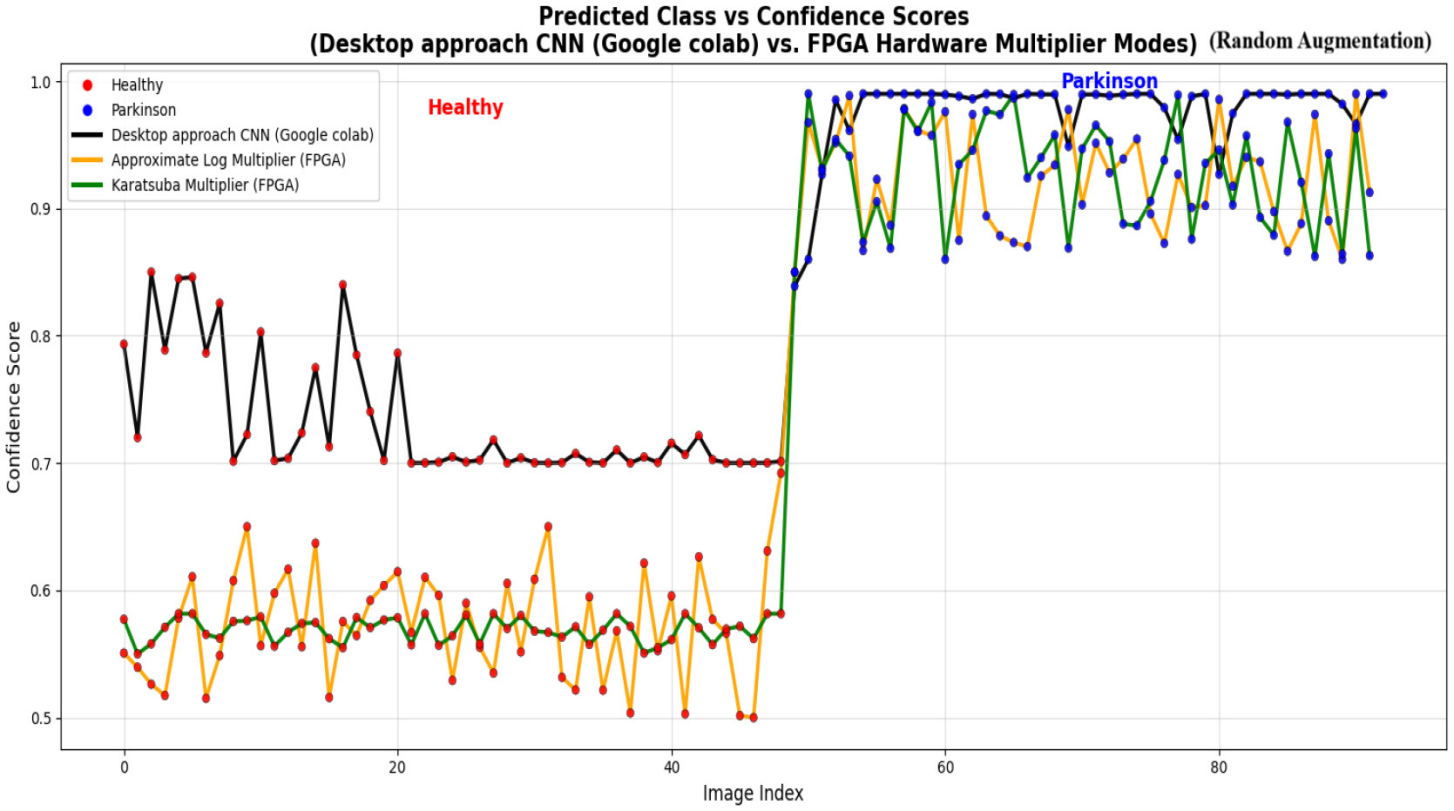
Comparison of predicted class confidence scores between the desktop approach CNN (Google Colab) and FPGA Hardware implementations using Approximate Logarithmic and Karatsuba multipliers (standard augmentation).

For each validation image, the predicted class label and corresponding confidence score obtained from the FPGA-based CNN accelerator were compared with the outputs of the desktop approach implemented in Google Colab for both the Standard Augmentation and non-augmentation validation methods. His graphical comparison plots evaluate the consistency between hardware and software inference and examine the impact of fixed-point quantization and multiplier approximation on the final classification. As shown in Figures 17, 18, the desktop CNN exhibits stable and well-separated confidence scores, with Healthy image samples typically producing confidence values in the range of (0.70–0.85) and Parkinson's samples yielding higher confidence values generally between (0.95–0.99). Both FPGA implementations successfully preserve this class-wise separation and follow the same confidence trends observed in the desktop CNN, indicating reliable transfer of the learned decision boundaries to hardware. Among the two FPGA designs, the Approximate Logarithmic Multiplier demonstrates the best confidence-score behavior for FPGA-based inference, showing closer alignment with desktop CNN outputs, smoother confidence variations, and reduced sample-to-sample deviation compared to the Karatsuba multiplier. Although both FPGA implementations maintain consistent predicted class labels across all validation images, the Karatsuba multiplier exhibits relatively higher fluctuations in confidence scores. These observations indicate that, from a confidence score perspective, the Approximate Logarithmic Multiplier more effectively preserves the CNN's decision behavior under fixed-point arithmetic, making it the preferred FPGA hardware multiplier architecture in this study.

### 7.1 Hardware synthesis and power analysis-FPGA

The trained desktop CNN model and its Verilog-based hardware implementation were synthesized and deployed on the Xilinx Zynq UltraScale+ ZCU106 evaluation board (XCZU7EV-FFVC1156-2-E) using Vivado Design Suite 2024.2, as shown in Table 11. The design operates at a clock frequency of 300 MHz and employs fixed-point (Q4.12) arithmetic, with all layer weights and biases preloaded into BRAM using (.mem) initialization files. Since both non-augmentation validation and Standard Augmentation approaches use the same CNN architecture and fixed-point configuration, the FPGA synthesis results (LUTs, FFs, DSPs, and power) remain unchanged. Data augmentation affects only the training process and does not influence the synthesized hardware implementation.

**Table 11:** Hardware implementation specifications of the proposed FPGA CNN accelerator.

Parameter	Specification
FPGA board	Xilinx Zynq UltraScale+ ZCU106
FPGA part number	XCZU7EV-FFVC1156-2-E
FPGA family	Zynq UltraScale+ MPSoCs
Design tool	Vivado Design Suite 2024.2
Operating frequency	300 MHz
Precision format	Fixed-point (Q4.12)
Total validation images	14

#### 7.1.1 Resource utilization

The post-synthesis utilization metrics for FPGA for various proposed multiplier configurations are presented in Table 12. The Approximate Logarithmic Multiplier demonstrates the lowest LUT (0.43%), FF (0.17%), and DSP (0%) consumption due to its simplified logarithmic-antilogarithmic arithmetic design. At the same time, the Karatsuba Multiplier offers a trade-off between computational performance and moderate resource utilization. The In-built Multiplier shows the highest resource usage, as it uses full-precision arithmetic and DSP-based computation.

**Table 12:** FPGA performance and resource utilization metrics for various proposed multiplier configurations.

S.No	Performance metric	In-built multiplier	Approx. log multiplier	Karatsuba multiplier
1	LUT usage	4,268 (1.96%)	924 (0.43%)	1010 (0.47%)
2	FF usage	5,357 (1.22%)	741 (0.17%)	991 (0.23%)
3	DSP usage	4 (0.22%)	0 (0%)	1 (0.20%)
4	RTL-level combinational propagation delay (ns)	214.00	151.67	189.59

The Approximate Logarithmic Multiplier achieves up to 78% reduction in LUT utilization and 83% reduction in FF utilization compared to the In-built multiplier while also demonstrating lower RTL-level combinational propagation delay. Among the evaluated multiplier architectures, the Approximate Logarithmic Multiplier provides the most resource-efficient implementation, making it particularly suitable for lightweight FPGA-based CNN acceleration in edge-oriented healthcare applications. The Karatsuba Multiplier provides intermediate results with balanced efficiency. The reported propagation delay values correspond to RTL-level timing measurements for the proposed multiplier-based CNN computation pipeline. They should not be interpreted as the complete end-to-end CNN inference latency. The total inference execution time depends on sequential layer execution, memory access, FSM scheduling, and the reuse of multiply-accumulate (MAC) computations across CNN layers.

#### 7.1.2 Power and performance analysis

Post-implementation power estimation and performance analysis are performed using Vivado's Power Analyzer tool in the Vivado Design Suite 2024.1. As shown in Table 13, the Approximate Logarithmic Multiplier achieves the lowest total power consumption (3.178 W) together with lower RTL-level combinational propagation delay, demonstrating improved hardware resource efficiency for FPGA-based CNN implementation. The Karatsuba Multiplier provides a balanced trade-off between power efficiency and computational precision. Conversely, the In-built Multiplier consumes the most power and occupies the largest area but maintains full arithmetic accuracy.

**Table 13:** Power and efficiency analysis for the proposed multiplier architectures.

S.No	Performance metric	In-built multiplier	Approx. log multiplier	Karatsuba multiplier
1	Power consumption (W)	29.517	3.178	23.174
2	Power efficiency (GOP/s/W)	3.47	388.5	42.62

The metrics are derived from Vivado's power and efficiency analysis results for the FPGA implementation.

1. Total Power (W):
$$\begin{array}{l} P_{\text{total}}=P_{\text{dynamic}}+P_{\text{static}} \end{array}$$ (11)
  Where:
  (a) P_dynamic-Power due to logic switching, routing, and signal activity.
  (b) P_static-Leakage power of FPGA resources.
  These values are obtained directly from the Vivado Power Analyzer, which estimates power after synthesis and place-and-route using switching activity files (.saif), as defined in Equation 11.
2. Power Efficiency (GOPs/W):
$$\begin{array}{l} \text{Power Efficiency}=\frac{\text{Throughput (GOP/s)}}{\text{Total Power (W)}} \end{array}$$ (12)
  The power efficiency is reported by the Vivado Power Analyzer and is used in Equation 12.
  Thus,
$$\begin{array}{l} {\text{Power Efficiency}}_{\left(\text{GOPs}/\text{W}\right)}=\frac{N_{\text{MAC}}}{T_{\text{exec}}\times P_{\text{total}}\times 10^{9}} \end{array}$$ (13)

The final Power Efficiency (GOPs/W) expression is given in Equation 13.

- The Approximate Logarithmic Multiplier achieves maximum efficiency (388.5 GOPs/W) due to its reduced arithmetic complexity and low dynamic power.
- The Karatsuba Multiplier demonstrates a balanced power-accuracy trade-off, maintaining higher precision while using reasonable energy.
- The In-built Multiplier is fully accurate but incurs the highest power consumption (29.517 W), resulting in the lowest efficiency (3.47 GOPs/W).

#### 7.1.3 Accuracy comparison-high-computation desktop approaches (Google Colab CNN) and HW FPGA

The classification accuracy of the proposed FPGA-based CNN accelerator was compared with that of a desktop CNN model trained and evaluated in Google Colab using 32-bit floating-point arithmetic. Conversely, the FPGA implementation performed inference using fixed-point Q4.12 quantization with two optimized multiplier designs. The comparative accuracy results are detailed in Table 14 for the non-augmentation validation and Table 15 for the Standard Augmentation non-augmentation validation approach, highlighting the performance difference between the desktop CNN (Google Colab) and FPGA-based hardware CNN.

**Table 14:** Accuracy comparison between desktop CNN (Google Colab) and FPGA-based CNN accelerator (non-augmentation validation).

Platform	Arithmetic type	Multiplier configuration	Accuracy (%)
Desktop CNN (Google Colab)	Float-32	None (baseline CNN)	92.86
FPGA (CNN accelerator)	Fixed-point Q4.12	Approximate logarithmic multiplier	89.73
FPGA (CNN accelerator)	Fixed-point Q4.12	Karatsuba multiplier	86.17

**Table 15:** Accuracy comparison between desktop CNN (Google Colab) and FPGA-based CNN accelerator (standard augmentation).

Platform	Arithmetic type	Multiplier configuration	Accuracy (%)
Desktop CNN (Google Colab)	Float-32	None (baseline CNN)	97.83
FPGA (CNN accelerator)	Fixed-point Q4.12	Approximate logarithmic multiplier	95.40
FPGA (CNN accelerator)	Fixed-point Q4.12	Karatsuba multiplier	94.70

For the non-augmentation validation approach (Primary evaluation methodology), the desktop CNN achieved an accuracy of 92.86%, while the FPGA implementations achieved 89.73% using the Approximate Logarithmic Multiplier and 86.17% using the Karatsuba Multiplier. Similar to the Standard Augmentation case, only a modest reduction in accuracy is observed due to fixed-point quantization.

For the Standard Augmentation approach, the FPGA-based implementations preserve most of the classification accuracy, showing only a slight reduction (approximately 2–3%) compared to the floating-point baseline desktop CNN (97.83%). This minor degradation is acceptable for real-time inference and helps suppress small floating-point variations, resulting in stable class separation during hardware execution.

Both hardware designs produce confidence score distributions that closely match each other, as indicated by the low Mean Absolute Deviation (MAD) values. Among the two configurations, the Approximate Logarithmic Multiplier consistently demonstrates the best trade-off between power efficiency and classification accuracy for PD detection on an FPGA.

## 8 Discussion

The comprehensive experimental results from both the high-computation desktop CNN model (Google Colab) and the FPGA hardware implementation demonstrate that the proposed CNN-based PD detection system is an energy-efficient and accurate architecture for lightweight edge-AI healthcare applications. The proposed approach successfully bridges the gap between software-level classification performance and hardware-level resource efficiency, positioning it as a suitable candidate for resource-constrained biomedical FPGA applications.

### 8.1 Software-level comparative analysis

Under the non-augmentation validation approach, the primary evaluation methodology, the proposed circle-based desktop CNN model trained on the NewHandPD dataset achieved a validation accuracy of 92.86%. In this approach, augmentation was applied exclusively to the training set and not to the validation set, thereby preventing augmented variants of the same original samples from appearing in both training and validation subsets and providing a more reliable evaluation of model generalization. Under the Standard Augmentation strategy, the proposed CNN achieved 99% training accuracy and 97.83% validation accuracy, with class-wise precision and recall of 100% for PD and 96% for healthy subjects.

To the best of our knowledge, Agrawal and Sahu (2024) are among the limited studies reporting results on the NewHandPD hand-drawn circle dataset, achieving an accuracy of 87.4% using VGG16 combined with Binary Gray Wolf Optimization and SVM. In comparison, the proposed FPGA-based CNN framework achieved validation accuracies of 89.73% under the non-augmentation validation approach, which serves as the primary evaluation methodology of this study, and 95.40% under the Standard Augmentation strategy. However, direct comparison across different studies remains challenging due to variations in dataset size, acquisition conditions, class balance, preprocessing methods, and evaluation protocols.

The Standard Augmentation approach yielded higher validation accuracy (97.83%), whereas the non-augmentation validation approach provided a more conservative and less biased estimate of model generalization performance by ensuring that no augmented or derived variants of the same original samples appeared in both the training and validation sets, thereby minimizing overlap between training and validation data. It should be noted that many prior studies rely on random splitting strategies without controlling for sample-level overlap, which may lead to optimistic performance estimates, whereas the proposed validation strategy enforces stricter evaluation conditions. However, since the validation subset contains only 14 samples, the reported validation accuracy is sensitive to individual misclassifications, with a single prediction error potentially changing it by approximately 7%. Therefore, the reported results should be interpreted with appropriate caution.

Overall, the proposed CNN provides a preliminary reference framework for circle-drawing-based Parkinson's handwriting classification by combining architectural compactness, fixed-point compatibility, and suitability for resource-efficient deployment on FPGA-based edge-AI healthcare research applications. The consistent performance observed across both validation strategies indicates stable behavior within the evaluated experimental settings.

In addition to efficient hardware deployment, recent advances in low-resource and data-efficient learning have demonstrated that competitive DL models can be developed using limited labeled data through strategies such as few-shot learning, active learning, transfer learning, and representative sample selection (Cao et al., 2025, 2023). While these approaches primarily focus on improving training efficiency, the proposed FPGA-based CNN framework addresses the complementary challenge of efficient inference acceleration for edge deployment. Integrating data-efficient training strategies with hardware-aware CNN deployment represents a promising direction for future resource-constrained edge-AI systems for PD diagnosis.

### 8.2 Hardware-level comparative analysis

The proposed CNN accelerator was implemented on the Xilinx Zynq UltraScale+ ZCU106 (ZU7EV) FPGA platform, which provides a balanced combination of LUTs, DSPs, and memory resources suitable for resource-constrained edge-AI inference. Both the Approximate Logarithmic Multiplier (ALM) and Karatsuba multiplier architectures were designed, synthesized, and evaluated on the same FPGA platform using the same CNN architecture and fixed-point Q4.12 representation. Based on this direct same-platform evaluation, the ALM-based implementation was selected for the final accelerator due to its more favorable trade-off between hardware resource utilization and computational efficiency for CNN workloads. Direct hardware comparison across different FPGA families, operating frequencies, precision formats, and application domains remains challenging due to variations in device architectures, workload complexities, and evaluation methodologies.

Therefore, the hardware-level discussion in this study primarily focuses on the implementation characteristics of the proposed CNN accelerator on the Xilinx Zynq UltraScale+ ZCU106 platform, including LUT utilization, flip-flop usage, DSP reduction, RTL-level combinational propagation delay, and power consumption. The ALM-based accelerator utilizes 924 LUTs, 741 flip-flops (FFs), and 0 DSP blocks, demonstrating a compact resource footprint suitable for lightweight FPGA deployment. Compared with the in-built multiplier implementation evaluated on the same FPGA platform, the ALM-based design achieves approximately 78% reduction in LUT utilization and 83% reduction in FF utilization while maintaining comparable classification functionality. The measured power consumption is 3.178 W, indicating the potential suitability of the design for low-power edge-oriented inference applications. Although the Karatsuba-based implementation also improves resource efficiency compared with the in-built multiplier architecture, the ALM-based design provides a more balanced trade-off among resource utilization, RTL-level combinational propagation delay, and power consumption for the evaluated CNN workload. Notably, both the non-augmentation validation and Standard Augmentation strategies use the same CNN architecture; therefore, FPGA hardware metrics, including LUT, FF, and DSP utilization, RTL-level combinational propagation delay, and power consumption, remain identical in both cases. The validation accuracies differ between the non-augmentation validation strategy (92.86%) and the Standard Augmentation comparative strategy (97.83%) due to differences in evaluation methodology rather than hardware implementation.

To the best of our knowledge, this study represents one of the early FPGA-based CNN accelerator implementations targeting PD hand-drawn circle classification using the NewHandPD dataset and optimized multiplier architectures.

Overall, the proposed FPGA-based CNN accelerator demonstrates the feasibility of lightweight fixed-point CNN deployment using optimized multiplier architectures for resource-efficient edge-AI healthcare research applications. However, due to differences in FPGA architectures, operating conditions, and evaluation methodologies, the presented hardware results should be interpreted primarily as a same-platform proof-of-concept implementation rather than as a universal benchmark across heterogeneous FPGA systems.

## 9 Conclusion

This study presents an FPGA-based hardware-software co-design framework for preliminary PD handwriting classification using hand-drawn circle images. The proposed system demonstrates the feasibility of resource-efficient CNN inference on FPGA hardware through deployment on the Xilinx Zynq UltraScale+ ZCU106 platform.

The key contributions of this study are summarized as follows:

1. A custom CNN architecture was developed and trained on the NewHandPD dataset using both non-augmentation validation and standard augmentation strategies. The primary evaluation methodology employed a non-augmented validation approach, achieving a software validation accuracy of 92.86% by applying augmentation exclusively to the training set and preventing overlap between training and validation samples. In comparison, the standard augmentation strategy achieved a validation accuracy of 97.83% and was used to compare augmentation-before-splitting effects.
2. The trained model was quantized to fixed-point Q4.12 representation and integrated into the Xilinx Vivado 2024.1 design flow using .mem-based deployment for FPGA realization. Three multiplier architectures—In-built Multiplier, Karatsuba Multiplier, and Approximate Logarithmic Multiplier (ALM)—were evaluated in the dense layer to analyze hardware performance trade-offs.
3. The ALM-based implementation achieved improved hardware efficiency, including significant reductions in LUT utilization, flip-flop consumption, DSP utilization, power consumption, and RTL-level combinational propagation delay.
4. Despite hardware-level optimization, the FPGA implementation maintained classification performance closely aligned with software inference, achieving validation accuracies of 89.73% under the non-augmentation validation approach. These results indicate the potential feasibility of approximate computing techniques for resource-efficient edge-AI healthcare applications.

Overall, the proposed FPGA-based CNN accelerator indicates the potential of lightweight hardware-aware DL frameworks for edge-oriented healthcare research applications. However, due to the limited size of the NewHandPD dataset, the presented findings should be interpreted as a preliminary proof-of-concept study rather than definitive clinical validation.

Future studies will focus on evaluation using larger and more diverse datasets, further optimization for ultra-low-power FPGA and ASIC implementations, and the investigation of video-based Parkinson's assessment, on-device adaptive learning, user-specific calibration strategies, and the integration of data-efficient learning with efficient pre-training, fine-tuning, and hardware-aware CNN deployment to further enhance resource-constrained edge-AI systems for PD diagnosis.

## Data Availability

The original contributions presented in the study are included in the article/supplementary material, further inquiries can be directed to the corresponding author.
